# Applications of SERS in the Detection of Stress-Related Substances

**DOI:** 10.3390/nano8100757

**Published:** 2018-09-25

**Authors:** Shuyuan Du, Chundi Yu, Lin Tang, Lixia Lu

**Affiliations:** 1 Shandong Provincial Key Laboratory of Animal Resistance Biology, Institute of Biomedical Sciences, Key Laboratory of Food Nutrition and Safety of Shandong Normal University, College of Life Science, Shandong Normal University, Jinan 250014, China; dusy@sdnu.edu.cn (S.D.); tanglin@sdnu.edu.cn (L.T.); 2 College of Food Science and Engineering, Qingdao Agricultural University, Qingdao 266109, China; naturalyu@qau.edu.cn

**Keywords:** SERS, stress, effector molecules, nanomaterials, trace-level analysis

## Abstract

A wide variety of biotic and abiotic stresses continually attack plants and animals, which adversely affect their growth, development, reproduction, and yield realization. To survive under stress conditions, highly sophisticated and efficient tolerance mechanisms have been evolved to adapt to stresses, which consist of the variation of effector molecules playing vital roles in physiological regulation. The development of a sensitive, facile, and rapid analytical methods for stress factors and effector molecules detection is significant for gaining deeper insight into the tolerance mechanisms. As a nondestructive analysis technique, surface-enhanced Raman spectroscopy (SERS) has unique advantages regarding its biosensing applications. It not only provides specific fingerprint spectra of the target molecules, conformation, and structure, but also has universal capacity for simultaneous detection and imaging of targets owing to the narrow width of the Raman vibrational bands. Herein, recent progress on biotic and abiotic stresses, tolerance mechanisms and effector molecules is summarized. Moreover, the development and promising future trends of SERS detection for stress-related substances combined with nanomaterials as substrates and SERS tags are discussed. This comprehensive and critical review might shed light on a new perspective for SERS applications.

## 1. Introduction

A wide range of stresses severely affect the growth and development of plants and animals worldwide. The stresses can be mainly divided into two broad categories: biotic stresses including different types of attacks from living organisms, (i.e., bacteria, fungi, viruses, nematodes and insects as well as other biology) [1,2,3,4]; abiotic stresses mainly caused by complex environmental conditions, (i.e., drought, submergence, salinity, hypoxia, heavy metal, pesticide and veterinary drugs, environmental organic pollutants, nutrient deficiency, light, UV, and extreme temperatures) [5,6,7,8,9,10,11]. To survive under stress conditions, highly sophisticated and efficient tolerance mechanisms are evolved to avoid, cope with, and adapt to all kinds of stresses, and have become a research hotspot.

**Scheme 1:**
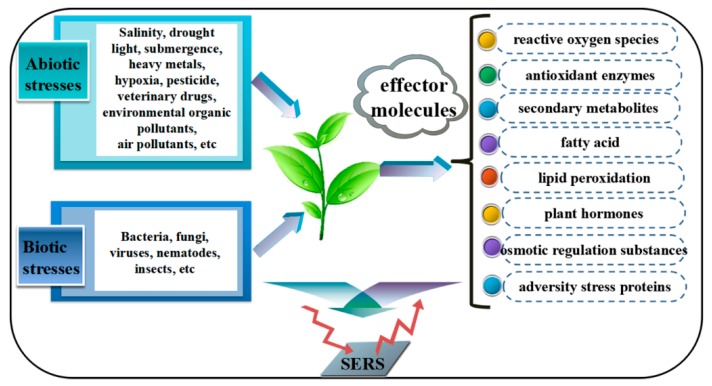
Abiotic and biotic stresses on the generation of effector molecules in plants.

Plants and animals share many tolerance mechanisms of unfavorable stresses. However, the mechanisms underlying the stress response in plants are probably more advanced and prominent than in animals [12]. During plant evolution, as sessile organisms, many mechanisms of stresses are activated at molecular, biochemical, physiological, and morphological levels [13]. In response to stress, a variety of effector molecules generated in plants, e.g., reactive oxygen species (ROS) [14], antioxidant enzymes [15,16,17], low molecular weight of secondary metabolites [18], unsaturated fatty acid and lipid peroxidation oxidation products [19,20,21], plant hormones [22], adversity stress proteins [23], chlorophyll and osmotic regulation substances [24] as shown in Scheme 1, have been investigated to study the tolerance mechanisms of biotic and abiotic stresses. Although many tolerance mechanisms of stress in animals similar to those in plants have been investigated, stress influences in the unique systems (cardiovascular, immune, and nervous systems) in animals still await discovery in detail [25]. Therefore, the stress factors and effector molecules in plants will be mainly described in this review.

These effector molecules play critical roles in the regulation of the plant growth, development, and responses to biotic and abiotic stresses. Therefore, the analysis of stress factors and effector molecules is very important for deeply exploring the mechanisms of response to unfavorable stresses and increasing yields to meet human demand. Various techniques have been established to detect trace stress factors and effector molecules, mainly including gas chromatography (GC), high-performance liquid chromatography (HPLC), mass spectroscopy (MS), atomic absorption spectroscopy (AAS), X-ray fluorescence spectroscopy (XPS), HPLC-MS, and GC-MS [26]. Even these methods are highly sensitive and selective, and are compromised with more expensive and larger instruments requirement and a labor-intensive sample handling and time-consuming detecting process, which hampers their practical application, especially in the field detection. Therefore, there exists increasing attention on exploring a sensitive, simple, and rapid technique for the detection of stress factors and effector molecules. Surface-enhanced Raman spectroscopy (SERS) can provide ultrahigh sensitivity and specificity from molecular fingerprint information and has been proved to be a universal analysis technique with great potential in the field detection, and extensively applied in quantification and imaging applications [27].

## 2. Principle of Surface-enhanced Raman Spectroscopy (SERS)

Since the discovery of Raman spectroscopy, due to the advantages of low regent consumption, high sensitivity and short response time, this technology has been widely used in many fields, such as medicine, archeology, gemstone identification, and materials [28]. It has great superiority in qualitative and quantitative analysis as well as molecular structure analysis of substances [29]. Furthermore, Raman spectroscopy has a series of characteristics such as rapidity, accuracy, simple sample pretreatment, and good reproducibility [30]. However, the conventional Raman scattering effect is extreme weak, whose scattering light intensity is only 1 × 10^−10^ of the incident light. Especially in the field for real-time detection, it is unlikely to achieve trace analysis of targets. Furthermore, it is not sensitive enough and, in most cases, requires a large integration time, potentially resulting in damage to the sample. As an extension and variation of standard Raman spectroscopy, SERS has emerged as a powerful analytical tool to solve the above problems. SERS refers to the phenomenon that the strength of the Raman scattering signal increases significantly when molecules or functional groups are adsorbed to specific surfaces of certain metals or semiconductors (such as the surfaces of nanoparticles, nanowires or rough surfaces with nanoscale roughness) [31]. When Raman spectroscopy is used to determine the target adsorbed on these special surfaces, the Raman signal can be enhanced by 10^14^–10^15^ times, which makes it possible to determine trace substances even down to single-molecule level.

Ever since the discovery of the SERS phenomenon, the selection and production of SERS substrate has always been the most critical technology, which not only determines whether SERS signals can be obtained, but also determines the quality of the SERS signal (including signal strength, stability, and repeatability). SERS mainly involves the use of the rough metal surfaces or metallic nanoparticles through special preparation. Optical properties of the metal nanostructures are the central topic of plasmonics. Different active substrates have different enhancement effects on the samples. The material itself, the shape and size of the nanoparticles all affect the enhancement efficiency of SERS [32]. Apart from the substrate, the SERS intensity is susceptible to many other factors, especially the distance between the signaling molecule (Raman dye) and substrate, as well as the variation of molecular structure.

However, the molecular mechanism of the SERS enhancement has not been clearly demonstrated. Typically, two primary mechanisms including chemical enhancement (CE) and electromagnetic enhancement (EM) are believed to be responsible, with the EM mechanism playing a predominant role [33,34]. The EM mechanism describes a purely physical effect concerning the enhanced local electromagnetic fields due to resonant excitation of plasma oscillations (plasmons) in the metallic nanostructure. In principle, the EM enhancement is chemically distance-dependent and nonselective. It offers similar enhancement for any type of molecules in the so-called “hot spot” that near the enormous enhanced filed with very close distances. Extreme field enhancements are only observed in hot spots such as the junctions between two particles, while sites experiencing moderate enhancement factors are much more frequent. However, this nonselective effect does not cover the direct interaction between the molecule and the underlying substrate. On the contrary, CE enhancement is selective and has been put forward to explain the interaction between the SERS substrate and chemisorbed molecules. This mechanism, which acts by virtue of the increase in the electronic polarizability of the adsorbate, is connected with the electronic properties of the adsorbed molecules. The charge transfer between the molecules in their electronic ground states and the metal which change the polarizability of the molecule contributes most to the CE effect and are widely accepted [35,36].

With respect to the detection of target analytes by SERS, generally, the detection modes can be divided into two categories: signal “turn-on” and signal “turn-off”, which refers to the SERS signal increases or decreases once targets exist in the sample. As a nondestructive analysis technique, SERS has unique advantages regarding its applications in biosensing. It not only provides a specific SERS fingerprint of the corresponding molecules, conformation, and structure, but also has multiplexing capacity for simultaneous detection and imaging of targets owing to the narrow width of the Raman vibrational bands. In the last few years, several new Raman spectrometers have been developed, ranging from large Raman spectrometers to miniaturized ones. Another attractive aspect of SERS method is its potential to be used in field detection. In the monitoring of the target analytes related to plant and animals, field detection is so intriguing that it is urgently needed. For this purpose, light, small and easy-to-use detection system is required. The commercialized hand-held Raman spectrometer characterized by miniaturization and portability has made on-site SERS detection a reality [37]. To achieve sensitivity improvement and space positioning, needle-tip enhanced Raman technology and confocal Raman technology have already appeared. The development and promising future trends of the SERS detection for stress-related substances are discussed in detail herein. Although many reported methods are not directly used to stress-related research, we believe this paper would enlighten researchers on the application of SERS in the study of plant and animal stress resistance. Meanwhile, it also can inspire new thoughts about SERS applications in the research of stress tolerance mechanisms, which includes the construction of substrates using new nanomaterials and the combination with other techniques.

## 3. SERS Applications in the Detection of Stress Factors

### 3.1. Inorganic Ions

#### 3.1.1. Heavy Metal Ions

Metals of those densities higher than 5 g·cm^−3^ are defined as heavy metals. With the urbanization, industrialization, automobile exhaust and intensive management of agriculture, heavy metal pollution, which is highly toxic to the growth and development of plants and animals, has attracted increased attention [38]. When organisms are exposed to heavy metals stress, detrimental effects would occur, such as the imbalance of ROS and the resistance to essential functional groups in biomolecules [39]. Among the common heavy metals, Fe, Zn, Ni, Cu, Co, and Cr are micronutrients but toxic at higher concentrations, whereas other metals such as As, Hg, Ag, Sb, Cd and Pb seem to be non-essential [40]. In contrast to the usual atomic spectrometry, SERS technique provides a superb platform for the detection of these metal ions, which are illustrated as follows.

Sun et al. [41] developed a high-performance SERS sensing platform for Hg^2+^ employing gold nanoparticles (AuNPs) modified silicon nanowire array as active substrate with signal “turn-on” strategy. Typically, single-stranded DNA would convert to the hairpin structure due to the formation of thymine (T)-Hg^2+^-T in the presence of Hg^2+^ ions, with strong SERS signals being detected. Similarly, using T base as the Hg^2+^ recognition molecule, Lu et al. [42] immobilized DNA aptamer which also contains the adenine and guanine bases on the surface of SiO_2_@Au core/shell nanoparticles. With specific interaction of Hg^2+^ with T bases in the aptamer, the DNA showed vertical orientation, leading to increase of Raman signal ratio I(660 cm^−1^)/I(736 cm^−1^), thus allowing the measurement of trace amounts of Hg^2+^ in the range of 0.1–1000 nM selectively. Makam et al. [43] reported a simple SERS marker using bolaamphiphile modified Au monolayers to provide a sensitive and selective detection platform for Hg^2+^, in which the fluorescence signal is also related to the concentration of Hg^2+^. Remarkably, this sensor offers an unprecedented Hg^2+^ detection limit of 60 × 10^−18^ M through SERS amplification technique. Determination of Hg^2+^ based on signal “turn-off” mode has also been developed. Silver nanoparticles (AgNPs) modified with sodium 2-mercaptoethanesulfonate showed strong SERS, while an intensity decrease of SERS is observed in the presence of Hg^2+^ due to the strong covalent bonding of mercury and sulfur [44]. Methimazole-functionalized and cyclodextrin-coated silver nanoparticles also serve as the SERS substrate for rapid and selective detection of Hg^2+^ [45].

In the list of 12 dangerous chemicals of global significance proposed by the United Nations Environment Program, cadmium was ranked first, which would interfere with erythropoiesis and thus cause anemic heart disease [46]. Yin et al. [47] presented a new design of “turn-on” SERS nanosensors for Cd^2+^ with AuNPs as the SERS-active nanoparticles, which are encoded with a new type of Raman reporter dye and a new polymer ligand to specifically chelate Cd^2+^. Addition of Cd^2+^ can lead to the inter-particle self-aggregation, accompanied by the SERS fingerprint signal increases with up to 90-fold. Such platform has potential to develop an array chip of nanoparticles to simultaneously detect multiple metal ions. Thatai et al. [48] compared the SERS sensitivities of AuNPs and core-shell SiO_2_@Au nanocomposites towards Cd^2+^ and confirmed that SiO_2_@Au was 20 times more sensitive than AuNPs with a limit of detection (LOD) of 100 ppb.

By taking advantage of DNAzyme’s specifically catalytic reacting with Pb^2+^, a SERS biosensor for sensitive detection of Pb^2+^ was proposed, with as low as 20 nM Pb^2+^ ions being detected. AuNPs nanozyme catalyzed the reaction of HAuCl_4_-H_2_O_2_ to form AuNPs rapidly that exhibited strong SERS effect, while Pb^2+^ aptamer adsorbed on AuNPs surface and inhibited its nanocatalysis. Upon the addition of Pb^2+^, it combined with aptamer to form a stable G-quadruplex, leading to the catalysis recovering and SERS intensity enhancing linearly [49]. Li et al. also presented similar sensor applying Pb^2+^ aptamer [50]. Besides, citrate functionalized gold nanoparticle [51], aminobenzo-18-crown-6 [52], the colloid of AuNPs/reduced graphene oxide [53] (rGO) were respectively used as the SERS substrates for selective determination of Pb^2+^. Yu et al. reported SERS chips for the simultaneous quantification of Pb^2+^ and Hg^2+^, with the combination of silicon nanohybrid as substrates and an internal standard sensing strategy [54].

Regarding the detection of As^3+^, silver nanomaterials were generally used as the SERS substrates in the reported literatures. Li et al. [55] presented a highly sensitive and selective SERS method for the trace analysis of As^3+^ ions using modified AgNPs, in which GSH was used to specifically bind with As^3+^ ions. The binding caused the aggregation of AgNPs, and satisfied Raman signal correlated with the concentration of As^3+^ was obtained. Ag nanoporous films [56] and Au@Ag core-shell nanoparticles [57] also acted as the SERS substrate for As^3+^ determination, respectively. Xu et al. [58] prepared a reproducible Ag nanofilm substrate taking advantage of a modified reaction for SERS detection of As^3+^ and As^5+^.

A plasmonic nanocomposities (AuNR@AuNPS-SERS nanotags/GO) was fabricated to establish a signal “turn-off” aptasensor for detection of Cu^2+^ [59]. A successful SERS sensor for Cu^2+^ detection was constructed based on gold nanostars coated with cysteine, which has strong coordination ability with Cu^2+^ and thus causing the aggregation of gold nanostars, along with distinct SERS signals being yielded [60].

SERS technique used for Zn^2+^ determination was also successful. Jin et al. [61] fabricated a SERS sensor platform based on 4-mercaptopyridine modified AgNPs which were anchored onto a silicon wafer, allowing for quantitative detection of Zn^2+^ ions.

#### 3.1.2. Anions

Apart from metal ions, SERS is also exploited for the determination and quantification of anion ions. Although the study of anionic stress is not as extensive as that of heavy metals, exposure to excessive anions also has a great impact on plants and animals. The primarily studied stress-related anions include Cl^−^ [62], NO_3_^−^ [63], SO_4_^2−^ [64] and so on. Analysis of these anions using SERS has also be reported. Although most of these methods are applied in environmental measurement [65,66,67], a large potential of SERS as a powerful tool has shown in the study of plant and animal stress tolerance. Moreover, Tran’s group exploited a self-designed Raman spectroscopy system aimed to get high signal-to-noise ratio, which may be worth learning by researchers [66].

### 3.2. Organic Pollutants

#### 3.2.1. Pesticides and Veterinary Drug Residues

Pesticides and veterinary drugs are commonly used to resist pests and diseases or to adjust the growth of plants and animals. As a kind of abiotic stress, their extensive use leads to serious environmental pollution and disturbance of various physiological processes in plants and animals [68]. Traditional chromatographic analysis for the detection of pesticides and veterinary drug residues are sensitive, accurate and reliable [69]. However, the rapid and efficient monitoring methods such as SERS for reliable and quantitative detection of such residues are still urgently required.

There are many studies about improving the sensitivity and reproducibility using excellent substrates and effective targets separation techniques in SERS [70]. Metal colloid-based substrates, such as Ag or Au nanomaterials, are most widely used as SERS nanosubstrates to improve the sensitivity [71]. However, the stability of these metal colloid-based substrates is susceptible to electrostatic repulsion. In addition, then metal-semiconductor hybrid nanomaterials with higher enhancement have been adopted [72]. Recently, there has been increased research interest in the positioning of solid surface-based substrates that influences the extraction of targets from complex surfaces. One type of solid SERS substrate is “hard” substrate, which immobilized small SERS-active building blocks on “rigid” substrates or films by complex and time-consuming methods. However, “flexible” polymer substrates consisting of plastics carbon fibers, plastics, papers, and adhesive tape are more effective for SERS detection [73]. A nanocomposite of Au nanofilms/cicada wing has been synthesized via a magnetron sputtering method and a LOD of 1 × 10^−9^ mg·mL^−1^ for acephate was acquired, which also could be applied to the rapid and sensitive detection of other trace residues [74].

An extraction and separation techniques are necessary in pesticide residues detection by SERS due to the complexity of sample matrices. However, much simpler sample preparations are enough for SERS detection compared to chromatographic methods [37]. He et al. proposed a SERS method for pesticide detection in apple peels based on a simple swab method [75]. In addition, a few studies on the detection of pesticide in apple peels adopting similar extraction procedure have also been reported, which demonstrated SERS can be applied successfully in detecting pesticides in lightly treated samples. In future, detection of the products of transformation and reaction, metabolites, impurities of pesticides and veterinary drugs during physiological activities of plants and animals using SERS should be emphasized and strengthened. The integration of SERS with other techniques can overcome limitations and bring more advantages such as result verification, better sample preparing procedures, and automating analysis. Another appreciable trend on the SERS detection of pesticides and veterinary drug residues is the potential to detect targets internalized in the cells of living tissues [73].

#### 3.2.2. Polycyclic Aromatic Hydrocarbons

Polycyclic aromatic hydrocarbons (PAHs) are widely distributed, toxic, highly carcinogenic, and environmentally persistent pollutants, which are combustion byproducts of carbon-based fuel [76]. The prior researches at the physiological and biochemical levels have shown that the oxidative stress and corresponding ROS are the main response to PAHs stress [77]. When the balance between pro-oxidants and antioxidant defenses (i.e., catalase CAT, superoxide dismutase SOD, glutathione peroxidase GPx and glutathione S-transferase GAT) is broken, the ROS and proteins levels in plants and animals would alter significantly [78]. Therefore, the ROS and antioxidase have been considered to be effector molecules that play vital roles in investigating the abiotic stress of PAHs [79] and will be elaborated later.

**Figure 1:**
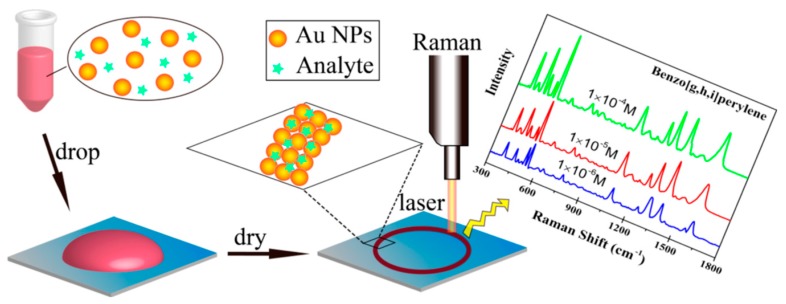
Facile detection of PAHs by a SERS based on the Au coffee ring effect. Reproduced from ref. [86], with permission from American Chemical Society, 2014.

An easy-to-operate, highly sensitive and selective SERS method has been proposed for PAHs detection to substitute traditional methods [15]. As nonpolar molecules, PAHs are poorly soluble in water and unsatisfied in affinity with bare metal surfaces. Therefore, functionalized Ag or Au substrates were first proposed to improve the adsorption of PAHs [80,81]. The functional molecules including thiol groups, calixarenes, and cyclodextrin derivatives have been adopted to increase the sensitivity of SERS for PAHs detection [82,83,84]. Recently, various novel SERS substrates with specific geometries and ultrahigh enhancement efficiency have been prepared for ultrasensitive PAHs detection as low as nM level, such as Au on TiO_2_ nanotube arrays, Au coffee ring (Figure 1) and Au on Fe_3_O_4_ or nickel 3D foam [81,85,86,87,88]. Au coffee ring effect refers to the accumulation of a dense, ring-like deposit on the border by evaporating a droplet of aqueous solution containing nonvolatile Au. Owing to the interplay of contact line pinning, solvent evaporation, and capillary flow of Au coffee ring, it is particularly suitable for material enrichment and material transport in SERS analysis [86]. The efficient and sensitive identification of multiple PAHs molecules based on single substrate is the biggest challenge of PAHs detection using SERS. Grafting AuNPs or AgNPs onto the surface of magnetic particles is another significant research progress to separate and enrich PAHs molecules before SERS detection [81].

#### 3.2.3. Formaldehyde

Formaldehyde, one kind of the abiotic stress factors, is a primary industrial chemical and a widespread environmental pollutant. A variety of changes in plants including chlorophyll content, the activity of peroxidase (POD) and ascorbate peroxidase (APX), carotenoid content, lipid peroxidation rate, the superoxide anion production rate and soluble protein and sugar contents would be caused by formaldehyde [89]. Meanwhile, formaldehyde can induce systemic toxicity by oxidative stress, which occurred in multiple tissues of formaldehyde-exposed rats and mice. The ROS is generated as the response to oxidative stress of formaldehyde, which can disrupt the physiological balance between oxidant and antioxidant enzymes [90].

Various analysis methods of formaldehyde have been explored, including colorimetry, fluorimetry, chemiluminescence, GC and HPLC [91,92,93]. Although their sensitivity, precision and accuracy are high enough for the trace detection of formaldehyde, the high-cost and large apparatuses are the challenges of on-site rapid screening. AgNPs as Raman-active substrates have been developed to detect formaldehyde based on the production of the Hantzsch reaction, which is a specific derivatization reaction between formaldehyde and 3-Methyl-2-benzothiazolinone hydrazone or 4-amino-5-hydrazino-3-mercapto-1,2,4-triazole (AHMT) [94,95,96]. However, using AuNPs as Raman-active substrates for formaldehyde analysis by SERS was scarcely studied. Lv et al. proposed a simple, controlled, and one-step scheme for synthesis of Au dendrites that served as Raman-active substrates for sensitive detection of formaldehyde based on the derivatization reaction of formaldehyde with AHMT [97]. Selection of suitable SERS substrates and derivatization reagents is the main direction to improve the SERS sensitivity and selectivity for the rapid formaldehyde analysis in real samples with complicated matrix.

### 3.3. Microorganism

#### 3.3.1. Bacteria

Higher plants are vulnerable to various forms of biotic and abiotic stress, among which bacterial infection is one of the main environmental stress factors encountered in the process of plant growth and development. Similar to plants, bacterial stress would cause various animal illnesses from minor skin infections to big life-threatening diseases. Common pathogenic bacteria include *Fusarium*, *Bacillus*, *Coccus*, *Vibrio*, *Salmonella* et al. [2,98,99,100,101,102,103]. Compared with typical methods such as plate counting and immunoassay, SERS technique has its unique advantages in the detection of pathogenic bacteria.

To capture target molecules more efficiently, aptamers instead of antibodies have been used for SERS assay of *Staphylococcus aureus*. Wang et al. [104] achieved single-cell detection of *S. aureus* through a magnetically assisted SERS biosensor with aptamer as the recognition element. They synthesized Ag-coated magnetic nanoparticles as the substrate and DTNB-labeled plasmonic NPs as novel SERS tags. Through optimizing the aptamer density and linker length, this assay can achieve excellent bacteria arrest (up to 75%). By replacing the corresponding aptamers, this SERS bioassay shows potential to monitor various bacterial pathogens and even cells. It can also be extended to the detection of multiple analytes if different aptamers are present. By the specific capture ability of aptamer, selective identification of *S. typhimurium* and *S. aureus* were realized based on magnetic AuNPs as substrate which was conjugated with aptamers of both *S. typhimurium* and *S. aureus* [105]. Except aptamers, vancomycin coating of plasmonic nanorod-array was used to capture *S. aureus* bacteria via multiple hydrogen bonding forces that existed between bacterial peptidoglycan and the carbonyl/amine groups of vancomycin. An ultralow SERS detection limit down to 17.8 cfu·mL^−1^ was achieved [106]. Lin et al. [107] embedded AuNPs in mesoporous silica with the aim of a high surface area for SERS though a convenient one-pot method and realized the detection of *S. aureus*. Regardless of typical and expensive SERS bases based on gold or silver, Kowalska et al. [108] chose bases that are fabricated using a simple and economic method through the decomposition of copper hydride.

Xu et al. [109] designed a sandwich structure using aptamers to quantify the concentration of *Vibrio parahaemolyticus*. This sandwich structure consisted of a first aptamer encoded on Fe_3_O_4_@Au/GO nanostructures to serve as the capture probe and a second aptamer modified with the Raman reporter molecule to undertake the SERS sensing task. This study can realize ultrasensitive detection of *V. parahaemolyticus* with a detection limit of 14 cfu·mL^−^^1^. They further designed a novel quasi-3D plasmonic nanostructure arrays to act as SERS-active substrates, which contribute to the rapid characterization and reliable quantification of different strains of *V. parahaemolyticus* on account of the SERS barcodes originated from the SERS spectra [110]. These unique SERS barcodes also offer the possibility of accurate identification of blind samples and mixtures.

**Figure 2:**
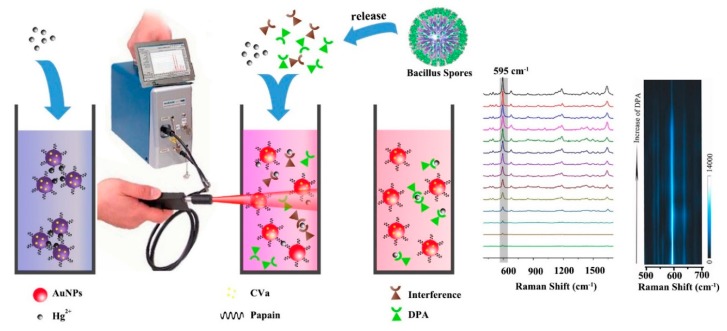
Schematic demonstration of Hg^2+^ ions aided SERS detection of DPA and SERS measurements. Reproduced from ref. [115], with permission from American Chemical Society, 2017.

*Bacillus anthracis*, generally known as anthrax, is classified as a class of dangerous bioterrorism agent. He et al. [111] proposed an innovative label-free platform for the recognition and detection of *B. anthracis* spores with silver dendrites as the SERS substrates, which was covered with aptamer for capturing the spores. Although the aptamer used was not specific enough against *B. anthracis* spores, the superior discrimination capacity of the SERS technology benefits the accuracy of this assay. Regarding the determination of *B. anthracis* by SERS, the more rapid and efficient approach is required to detect dipicolinic acid (DPA), which serves as an excellent biomarker of anthrax. Lu’s group [112] firstly achieved a LOD of about 4 × 10^−6^ M CaDPA based on SERS-active silicon nanowire arrays coated with silver. Farquharson’s group [113] made use of ATYPLPIR, a peptide having stronger affinity to *B. anthracis* than other species of *Bacillus*, to offer the SERS assay with 10^4^ spores per mL every 10 min. Cheung et al. [114] proposed a SERS method composed of meso-droplets on superhydrophobic wires and achieved the sensitive detection of DPA, well below the infective dose. Based on the interaction between DPA and Hg^2+^, rapid and ultrasensitive determination of DPA with zero-background was reported. As shown in Figure 2, the introduction of Hg^2+^ and DPA induced the controllable aggregation of papain-capped AuNPs, which initiated the variation of SERS intensity of cresyl violent acetate conjugated AuNPs correspondingly. The depressed Raman intensity is linearly dependent on the concentration of DPA in the range of 1 nM-8 μM, with an extremely low detection limit of 67.25 pM [115]. Furthermore, field analysis would be realized using portable Raman spectrometer.

#### 3.3.2. Virus

Viruses are small agents that can infect life forms from animals to plants and replicate inside the living cells of other organisms. There are various viruses widely found in animals, such as bovine ephemeral fever virus [4,116], bovine rhinotracheitis virus [117], Newcastle disease virus [118], respiratory syndrome virus [119], poly (I:C) [120,121,122], porcine circovirus [123] and so on, which cause infectious diseases and an enormous economic loss. Thus, it is significant to develop sensitive and facile methods for detecting these viruses to handle the infectious diseases. As a rapid and nondestructive technique, SERS has come to be applied in virus identification and these reported sensors would provide an enlightening idea to the recognition of a variety of viruses.

Au/Ag multilayered nanorod arrays was fabricated via the focused ion beam technique as SERS substrates for the identification of the influenza A virus strain with a detection limit of 10^6^ PFU·mL^−1^ [124]. Lim et al. [125] proposed a SERS assay for in situ influenza virus identification. This assay successfully distinguished four different viruses on the hypothesis that every newly emerging virus possesses unique surface lipids and proteins, which generated their own characteristic Raman spectra by interacting with gold nanostructures. Sun et al. [126] demonstrated a magnetic immunosensor for avian influenza virus detection based on Fe_3_O_4_/Au NPs as both capturing and supporting substrates with high SERS activity. This sensor showed excellent sensitivity with a lowest concentration of H3N2 H_3_N_2_ down to 1 × 10^2^ TCID_50_·mL^−1^. Porcine circovirus (PCV) is the first and smallest animal virus. A facile immunoassay for PCV2 using multi-branched AuNPs as SERS substrates was also proposed [127]. Prion proteins, which can infect animals, were quantified by a SERS-based optical platform making use of its higher binding affinity for Cu^2+^ ions [128].

### 3.4. Air Pollutants

Air pollutants are crucial sources of abiotic stress on plants, which primarily includes carbon oxides, nitrogen oxides, ozone, sulfur oxides and ammonia. When leaves are chronically exposed to the air pollution, the waxy layer that protects plants from water loss, drought diseases, pests, and frost would be damaged, and many physiological processes in plants would be modified.

Ozone, a phytotoxic gaseous air pollutant, has severe effects on plant and is considered to be a significant threat to crop production [129]. Ozone enters mesophyll tissue through stomata and then produces ROS, which can damage the cell membrane, accelerate the lipid peroxidation, lead to chlorophyll degradation, decrease the photosynthesis rate, and depress biomass accumulation [130,131]. For developing a sensitive and selective detection method of ozone, SERS has been proposed and practiced in air samples. Zhang et al. took rhodamine S as a molecular probe on a substrate of aggregated AuNPs sol and realized trace ozone monitoring with a LOD of 0.9 nmol·L^−1^ [132].

The rapid growth of the anthropogenic production of carbon dioxide (CO_2_) has opened serious issues related to global warming and abiotic stress on plants. CO_2_ dissolved in groundwater can increase the concentration of aqueous carbonate species and cause significant changes in pH. Therefore, the viability and activity of many species, such as green alga microorganisms have been influenced by dissolved CO_2_ [133,134]. Employing SERS as a new strategy for capturing and monitoring CO_2_ has been proposed. Lust et al. has demonstrated that the SERS spectra of CO_2_ adsorbed on cold-deposited copper films could be clearly displayed [135]. SiO_2_/TiO_2_, core/shell beads, have been designed and fabricated for SERS detection of CO_2_ under real working circumstances [136].

Nitrogen oxide, sulfur oxides and ammonia, the composition of air pollution, can also cause abiotic stresses that influence a variety of physiological functions and the defense responses in plants and animals. However, until now very few researches on the application of SERS have been proposed for detection of these ingredients.

## 4. SERS Applications in the Detection of Effector Molecules

Abiotic and biotic stresses on plants and animals are important limiting factor to their growth, development, reproduction, and yield realization. Plants and animals share some tolerance mechanisms to unfavorable stress. However, plants without a well-defined immune system have much more complex response system to stress and are mainly described here. A mass of effector molecules would be generated in plants to respond to stress. Therefore, monitoring them is beneficial to investigating the tolerance mechanisms of plants. Therefore, the SERS applications in the detection of effector molecules in plants will be reviewed in this part.

### 4.1. Reactive Oxygen Species (ROS)

ROS, such as H_2_O_2_ and superoxide anion radical (O_2_^−^), are physiological metabolites in plants that play a significant role in cell signal transduction, growth, development, and biotic or abiotic stress responses [137]. When plants are subjected to stress, a large amount of ROS is produced in the body, disrupting the ROS homeostasis and causing oxidative damage to membrane lipids [138]. Besides, ROS play a vital role as signaling molecules. In consequence, the accurate measurement of ROS is indispensable for the deep understanding of stress tolerance and regulation.

Using the reducibility of H_2_O_2_, AuCl_4_^−^ was reduced to Au^0^ that enlarge the AuNPs adhered to the surface of SiO_2_. With the increase of H_2_O_2_ concentration, the resultant gold on the surface of silica cores keep growing correspondingly until continuous gold nanoshells come into being. During the process, the SERS activity was strongly correlated with the amount of H_2_O_2_, which allows the quantification of H_2_O_2_. Furthermore, H_2_O_2_ scavenging activity was also determined [139]. Except gold, silver is also used as SERS substrate for H_2_O_2_ detection. Chen et al. [140] reported a SERS and electrochemical dual sensor for H_2_O_2_ that was fabricated based on silver nanowires deposited on coffee filter through a simple dip-coating route. They also deposited silver nanowires on fluorine-doped tin oxide glass which functioned as both SERS substrate and electrochemical sensor for H_2_O_2_ [141]. Qin et al. [142] constructed AuNPs/dopamine sensing platform. The SERS signal differences between AuNPs/dopamine and AuNPs/dopamine-quinone indicated the platform showed great potential for the identification of GSH or O_2_^−^ with high sensitivity. Above all, when being introduced into living cells, the AuNPs/dopamine platform could realize the real-time delivery of ROS information. Dong et al. [143] proposed a free radical-quenched SERS probe using starch to coat gold nanoshells that formed a protective layer as the enhancement substrate. Methylene blue, which served as the signal molecule, was then adsorbed on starch-coated gold nanoshells. Methylene blue can react with free radical converted from H_2_O_2_ to quench its SERS signal, thus the detection of H_2_O_2_ was realized. This probe can also be used for the detection of glucose since it can be transformed to H_2_O_2_ in the presence of glucose oxidase.

### 4.2. Enzymatic Antioxidants

As byproducts of different biotic and abiotic stresses, the ROS as the first layer of defense accumulate quickly in organelles such as chloroplasts, peroxisomes, mitochondria plasma membrane and cell wall [144]. Then the oxidative stresses induced by ROS cause a series of changes in cell including carbohydrate, protein, lipid, and nucleic acid [145,146,147,148,149]. The excessive ROS is counter balanced by antioxidative defense system including enzymatic and nonenzymatic antioxidants. The enzymatic antioxidants mainly include APX, CAT, SOD, POD and GR [138,150,151,152,153]. Secondary metabolites antioxidants, the nonenzymatic antioxidants, such as glutathione, chlorophylls, ascorbic acid, carotenoids, and tocopherols are involved in ROS quenching [150,153,154,155].

Avoiding the time-consuming sample preparation and large apparatuses needed by traditional detection methods, SERS have been employed to the rapid and sensitive detection of antioxidant enzyme [156,157]. Horseradish peroxidase (HRP) and its activity have been measured by single-molecule SERS through polymer-bridging flocculation to aggregate the AgNPs [158]. The SERS spectra from the assembled AgNPs/protein films showed excellent reproducibility and sensitivity regardless of the charge status and size of enzyme [159]. Silver nanocrystallite based on porous silicon has been used as efficient SERS substrate for trace HRP detection [160]. Cottat et al. used gold nanoantennas to develop a novel SERS nanobiosensor with high specificity and sensitivity offered by thiolated aptamers for MnSOD detection [161]. Although a few SERS methods have been reported for antioxidant enzyme detection, there are still enormous requirements to establish sensitive, rapid, and simple detection methods.

### 4.3. Nonenzymatic Antioxidants

Chlorophylls, phenols, flavonoids and the like, which are secondary metabolites antioxidants, are mainly monitored by spectrophotometer and chromatographic techniques [138,162]. An economical and rapid SERS has also been proposed recently. Lian et al. employed SERS to detect the chlorophyll in vegetable oils, which does not require any sample pretreatment and greatly shorten the testing time [163]. Ferulic acid, p-coumaric acid, sinapic acid and caffeic acid are typical examples of important natural phenolic antioxidants. Aguilar-Hernández et al. [164] prepared diverse SERS-active silver colloids and clustered the as-obtained colloids employing Principal Component Analysis (PCA) based on the concentration and nanoparticle size, to systematically evaluate these phenolic antioxidants by SERS measurements sensitively. In general, flavonoids possess multiple OH groups, which may interact with AgNPs and play a part in the activation of SERS. Therefore, SERS spectra of various flavonoids were mostly investigated in the presence of AgNPs [165]. Taking advantage of the aggregation of citrate-capped AuNPs, Huang et al. [166] reported a SERS sensor for the analysis of catechin, a kind of natural flavonoid associated with a myriad of biological effects.

### 4.4. Plant Hormones

Plant hormones are a group of naturally occurring trace organic compounds synthesized in plants [167,168]. Plant hormones as chemical messengers play vital roles in the regulation of physiology during plant growth and development, as well as in response to various biotic and abiotic stresses [169,170,171,172,173]. To date, nine recognized types of plant hormones including auxins (IAA), cytokinins (CTK), gibberellins (GA), ethylene (ETH), abscisic acid (ABA), brassinosteroids (BR), jasmonates (JA), salicylic acid (SA) and strigolactones (SL) have been identified and play crucial roles in response to stress in plants [174,175,176]. Nitric oxide (NO) [169], cytochrome P450s [177], protein kinase [178] and polypeptide are characterized as plant hormone analogues due to their similar modes of physiological action. Their occurrence and content depend strongly on the plant organ, plant age, developmental stage, environmental conditions, and stresses [179].

**Figure 3:**
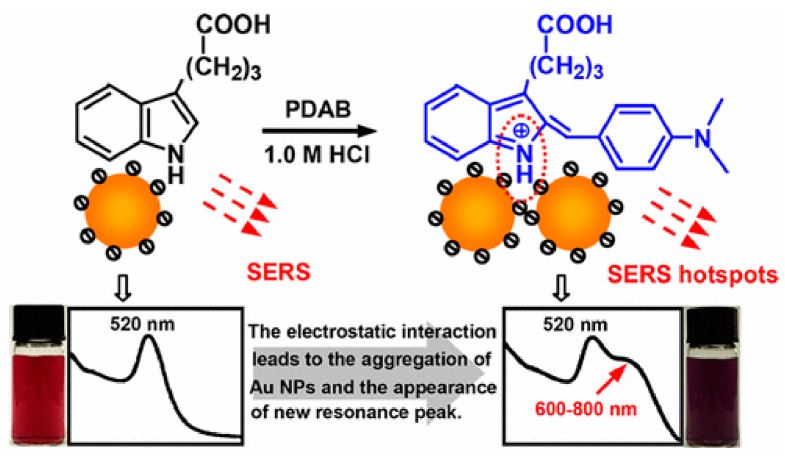
Combination Ehrlich reaction with AuNPs for ultrasensitive detection of indole-3-butyric acid. Reproduced from ref. [185], with permission from American Chemical Society, 2017.

Plant hormones, small molecular secondary metabolites presented in plant tissues, with high bioactive signaling and extremely low concentrations (generally 1 μmol·L^−1^) regulate all plant developmental processes [180]. However, plant hormones are easy to be inactivated due to their unstable properties. Meanwhile, plant tissues are complicated multicomponent mixtures containing trace plant hormones and other compounds similar to hormones in structural and/or physicochemical properties. Therefore, rapid and reliable analytical methods are urgently required. The currently mainstream methods of sensitive determination of plant hormones, such as ELISA, HPLC, GC/MS, LC/MS, and flow injection fluorimetry are time-consuming and require complicated sample preparation and enrichment [181,182]. SERS with high sensitivity and unique spectroscopic fingerprint, has been greatly applied as a useful tool for the analysis of trace plant hormones in complex matrix using noble metal nanoparticles. N^6^-Benzylademine, a kind of cytokinins, has been proved to control plant adaptation to biotic and abiotic stresses. N^6^-Benzylademine with the protective effects on the growth, photosynthesis, and antioxidant capacity in the plant leaves plays a significant role in the tolerance of plants to multiple stresses such as salinity, drought, submergence, pest and disease and extreme temperatures [183]. A rapid and sensitive SERS for the analysis of trace N^6^-Benzylademine in complicated matrix has been established using the gold nanoparticle colloid substrate [184]. Indole-3-butyric acid is a classic plant hormone that plays a vital role in promoting the root development to tolerate stresses of plants. Combination Ehrlich reaction, transforming indole-3-butyric acid into a Raman-active resonant molecule, has been proposed for ultrasensitive detection of indole-3-butyric acid, showed in Figure 3 [185]. Brassinosteroids, a kind of polyhydroxy steroids, can regulate plant growth, development, and physiological phenomena at very low concentrations. Chen et al. has developed a novel label-free AuNPs-immobilized paper strip with SERS effects for trace detection of brassinosteroids. Which was fabricated using poly (γ-glutamic acid) as the linker and concentrator for immobilizing AuNPs [186]. The prospects for the detection of plant hormones should include the quantification of multiple kinds of targets simultaneously. Development of sensitive, transient, in situ, and dynamic analytical technique for trace detection of plant hormones in a live plant is another challenge.

NO has been commonly known as a source of air pollution due to the contribution to acid rain and the damage of the ozone layer. However, NO also play important roles in a wide variety of physiological functions such as plants hormones in plants, such as seed germination and dormancy, root growth, flowering, senescence, and tolerance to abiotic stress [25,187]. Meanwhile, NO also participates in a broad spectrum of functions in animals [25]. Most of traditional assays are not suitable for the direct detection of trace NO in living system. Because of important advantages of provision of fingerprinting information and resistance to photo-bleaching, SERS has been widely applied to monitoring NO in complex matrices. Integrating Raman reporter molecules with SERS-active nanostructures reacting with NO could induce changes of SERS spectra. Cui et al. employed o-phenylenediamine-modified AuNPs as nanoprobes to detect the NO in living cells [188]. A ratiometric SERS probe based on immobilizing 3,4-diaminobenzene-thiol onto trisoctahedral gold nanostructures has been successfully developed for real-time monitoring and imaging of trace NO in live cells [189]. Then monitoring of NO released from an individual bacterium in situ has been successfully realized adopting plasmonic nanostructure-based live bacteria as SERS platform [190]. Recent research reported a molecular probe based on bodipy that can systematically provide temporal information on NO by fluorescence imaging and SERS fingerprinting [191].

### 4.5. Production of Lipid Peroxidation

The structure and fluidity of plant cell membrane are affected by lipid composition and the variable unsaturated fatty acid level. Fatty acids are key components of membrane lipid and are precursors to several signaling and defense compounds [20]. Many reports have indicated that the increase of unsaturated fatty acid played a critical role in the protection of the photosynthetic apparatus from various stresses, especially heat, cold, drought and salt stress [19,192,193]. Moreover, the increase of ROS induced by stresses has been recognized for the membrane lipid peroxidation. Malondialdehyde (MDA) and arachidic acid, the decomposition products of the oxidation of fatty acid, has been used as an indicator of lipid peroxidation [138,194,195]. Monitoring the level of fatty acid and MDA is an effective way to investigate lipid peroxidation and tolerance mechanisms in plants.

GC, GC-MS and HPLC are routine methods for determination of individual fatty acid content [196,197]. As a useful analysis tool, SERS has been applied to the sensitive and selective detection of fatty acid adsorbed on metal nanostructures. A simple, large-active-area SERS substrate, gold-coated horizontally aligned carbon nanotube (Au-HA-CNT), has been fabricated and suitable for trace fatty acid analysis [198]. The SERS spectra obtained from Ag hydrosol/DMTAP system has been used for cationic lipids detection [199]. New SERS substrates that are large-scale two-dimensional arrays of metal nanostructures derived from thin-film evaporation over polystyrene spheres have been established and combined with atomic force microscopy for topographical imaging and tracing arachidic acid [200].

The spectrophotometric and fluorescence are main detection methods of MDA, which are based on highly chromogenic and fluorescent TBA-MDA adduct [3,152,201,202,203]. Due to the poor specificity of spectrophotometric and fluorescence feature of TBA-MDA, the sensitivity and specificity of spectrophotometric methods are not enough in the quantification of the lipid peroxidation. Zhang et al. used AgNPs as the substrates to monitor lipid peroxidation by detecting TBA-MDA adduct [204].

### 4.6. Osmotic Regulation Substances

Under stress conditions, such as salt, cold and drought, osmotic stress would be induced in plant cells, and reestablishing osmotic equilibrium is extremely essential for plants. There are usually two ways for osmotic regulation. One is to absorb and accumulate inorganic salt ions; another primary strategy is the synthesis and accumulation of compatible osmolytes, which are mainly small organic molecules, including proline [205], glycine betaine [206], and soluble sugar (such as glucose and fructose) [207,208] to lower the osmotic potential in the cell. Given all that, monitoring the contents of these small molecules is very necessary to gain deep insight into the mechanism of plant stress responses and tolerance. SERS technique would serve as a very powerful tool to achieve this goal.

The SERS spectrum of L-proline was recorded and studied combining the theoretical analysis based on a silver surface. The interaction between L-proline and metal was also analyzed [209]. Cárcamo and co-workers [210] obtained SERS spectra of proline and hydroxyproline using colloidal Ag acquired by reduction with hydroxylamine.

**Figure 4:**
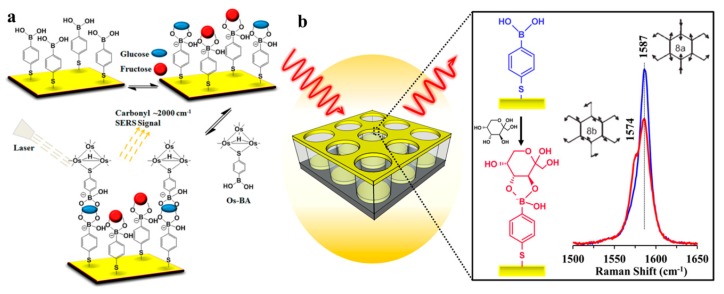
A glucose molecule brings Os-BA to the substrate via formation of a bidentate complex (**a**); Schematic illustration of 4-MPBA on a gold Q3D-PNA for the detection of fructose using SERS (**b**). Reproduced from ref. [211,213], with permission from American Chemical Society, 2013 and 2014, respectively.

Kong et al. [211] have demonstrated a SERS assay for glucose based on the conjugation of triosmium carbonyl cluster and boronic acid (Os-BA), as shown in Figure 4a. Using the stretching vibrations of CO in the metal carbonl for SERS quantification, this assay is free from conjugation of SERS-active species. Making use of the specific binding between 4-mercaptophenylboronic acid (4-MPBA) decorated on AgNPs and glucose, a simple “turn-on” SERS nanosensor was designed [212]. Using the similar principle, 4-MPBA modified on the gold surface was also chosen as the probe molecule for the detection of fructose by SERS technique [213], as illustrated in Figure 4b. In fact, most sensors for glucose are based on the detection of H_2_O_2_ that was produced from the oxidation of glucose catalyzed by GO*x*. Qi et al. [214] have designed a facile and sensitive “turn-off” SERS sensor for glucose using the etching effect of AgNPs by H_2_O_2_ derived from glucose with the existence of GO*x*. The SERS signal of 4-mercaptopyridine as Raman tags marked on AgNPs decreased along with the etching of AgNPs. The lowest concentration that can be detected is 10 μM. Gu et al. [215] reported a SERS probe for H_2_O_2_ with a LOD of 70 nM based on 3-mercaptophenylboronic acid modified AuNPs. In addition, this probe can further be coupled with GO*x* to achieve selective determination of glucose. Fu et al. [216] presented a glucose-detecting SERS sensor based on a sandwich structure composed of gold wafer assembled with 4-aminothiophenol and the Ag+-mediated combination of AgNPs marked with cysteamine through the coordination bond of N→Ag^+^←N. In addition, here, Ag^+^ ions come from the etching of AgNPs by H_2_O_2_ arising from the oxidation of glucose. Apart from gold and silver-based substrates, Cai et al. [217] immobilized Pt nanoparticles on TiO_2_ nanotube array electrochemically as SERS reversible substrates for the construction of nonenzymatic glucose sensors.

### 4.7. Others

Changes in concentration, type, and activity of trace effector molecules such as glutathione and protein play critical roles in regulatory mechanisms to adapt to a variety of stresses [23,154]. The SERS substrate based on the AgNPs substrate has been adopted for detecting trace stress proteins HSP70. Only a two-step process for HSP70 detection was needed, which is much simpler compared to the multi-step ELISA method and has the potential to replace ELISA [218]. AgNPs prepared through the reduction of AgNO_3_ by beta-cyclodextrin were employed to achieve sensitive SERS analysis of glutathione [219]. These researches allowed us to suggest that SERS would develop into a promising technique for simple, rapid, and on-site screening of trace effector molecules for deeply exploring the tolerance mechanisms.

## 5. Conclusions and Perspective

SERS provides ultrahigh sensitivity and specificity due to molecular fingerprint information and has been proved to be a powerful analysis technique extensively applied in many fields. Although SERS has been successfully used for the sensitive and specific detection of trace stress factors and effector molecules, there are still plenty of challenges that need to be addressed. For instance, biocompatible, stable, inexpensive, and reliable substrates using new nanomaterials with uniformly high enhancements should be exploited. In addition, the integration of SERS with other techniques cannot only broaden its usage scope, but also bring about more advantages such as result verification, better sample preparing procedures, and automating analysis. Another prospect for investigating tolerance mechanisms using SERS should include the quantification of multiple targets simultaneously and the development of transient, in situ, and dynamic analytical methods for trace target detection in live plants and animals. With further development, SERS shows great potential to be a robust and reliable analytical technique for intensive studying in tolerance mechanisms.

## References

[1] Li L., Yang H.J., Liu D.C., He H.B., Wang C.F., Zhong J.F., Gao T.D., Zeng Y. (2012). Analysis of biofilms formation and associated genes detection in staphylococcus isolates from bovine mastitis. Intern. J. Appl. Res. Vet. Med..

[2] Huang Y.H., Wang X.J., Zhang F., Huo X.B., Fu R.S., Liu J.J., Sun W.B., Kang D.M., Jing X. (2013). The identification of a bacterial strain BGI-1 isolated from the intestinal flora of *Blattella germanica*, and its anti-entomopathogenic fungi activity. J. Econ. Entomol..

[3] Song J., Shi W., Liu R., Xu Y., Sui N., Zhou J., Feng G. (2017). The role of the seed coat in adaptation of dimorphic seeds of the euhalophyte *Suaeda salsa* to salinity. Plant Spec. Biol..

[4] He C.Q., Liu Y.X., Wang H.M., Hou P.L., He H.B., Ding N.Z. (2016). New genetic mechanism, origin and population dynamic of bovine ephemeral fever virus. Vet. Microbiol..

[5] Ashrafi-Dehkordi E., Alemzadeh A., Tanaka N., Razi H. (2018). Meta-analysis of transcriptomic responses to biotic and abiotic stress in tomato. PeerJ.

[6] Zhang T., Song J., Fan J., Feng G. (2015). Effects of saline-waterlogging and dryness/moist alternations on seed germination of halophyte and xerophyte. Plant Spec. Biol..

[7] Tian W., Zhang H.Y., Zhang J., Zhao L., Miao M.S., Huang H. (2017). Responses of zooplankton community to environmental factors and phytoplankton biomass in Lake Nansihu, China. Pak. J. Zool..

[8] Song J., Shi G., Gao B., Fan H., Wang B. (2011). Waterlogging and salinity effects on two *Suaeda salsa* populations. Physiol. Plant..

[9] Zhang X.Y., Lou M.F., Shen W., Fu R.S., Wang D.H. (2017). A maternal low-fiber diet predisposes offspring to improved metabolic phenotypes in adulthood in an herbivorous rodent. Physiol. Biochem. Zool..

[10] Yuan F., Chen M., Yang J., Song J., Wang B. (2015). The optimal dosage of ^60^co gamma irradiation for obtaining salt gland mutants of exo-recretohalophyte *Limonium bicolor* (Bunge) O. Kuntze. Pak. J. Bot..

[11] Cheng S., Yang Z., Wang M., Song J., Sui N., Fan H. (2014). Salinity improves chilling resistance in *Suaeda salsa*. Acta Physiol. Plant..

[12] Qin F., Shinozaki K., Yamaguchi-Shinozaki K. (2011). Achievements and challenges in understanding plant abiotic stress responses and tolerance. Plant Cell Physiol..

[13] Perez-Clemente R.M., Vives V., Zandalinas S.I., Lopez-Climent M.F., Munoz V., Gomez-Cadenas A. (2013). Biotechnological approaches to study plant responses to stress. Biomed. Res. Int..

[14] Sui N. (2015). Photoinhibition of *Suaeda salsa* to chilling stress is related to energy dissipation and water-water cycle. Photosynthetica.

[15] Zhao S.Z., Sun H.Z., Chen M., Wang B.S. (2010). Light-regulated betacyanin accumulation in euhalophyte *Suaeda salsa* calli. Plant Cell Tissue Org. Cult..

[16] Zhao S.Z., Sun H.Z., Gao Y., Sui N., Wang B.S. (2011). Growth regulator-induced betacyanin accumulation and dopa-4,5-dioxygenase (*DODA*) gene expression in euhalophyte *Suaeda salsa* calli. In Vitro Cell. Dev. Biol. Plant.

[17] Sui N., Tian S., Wang W., Wang M., Fan H. (2017). Overexpression of glycerol-3-phosphate acyltransferase from *Suaeda salsa* improves salt tolerance in arabidopsis. Front. Plant Sci..

[18] Haak D.C., Fukao T., Grene R., Hua Z., Ivanov R., Perrella G., Li S. (2017). Multilevel regulation of abiotic stress responses in plants. Front. Plant Sci..

[19] Liu S., Wang W., Li M., Wan S., Sui N. (2017). Antioxidants and unsaturated fatty acids are involved in salt tolerance in peanut. Acta Physiol. Plant..

[20] Sui N., Wang Y., Liu S., Yang Z., Wang F., Wan S. (2018). Transcriptomic and physiological evidence for the relationship between unsaturated fatty acid and salt stress in peanut. Front. Plant Sci..

[21] Ahmad P., Jaleel C.A., Salem M.A., Nabi G., Sharma S. (2010). Roles of enzymatic and nonenzymatic antioxidants in plants during abiotic stress. Crit. Rev. Biotechnol..

[22] Yuan F., Chen M., Yang J., Leng B., Wang B. (2014). A system for the transformation and regeneration of the recretohalophyte *Limonium bicolor*. In Vitro Cell. Dev. Biol. Plant.

[23] Zhang J.X., Wang C., Yang C.Y., Wang J.Y., Chen L., Bao X.M., Zhao Y.X., Zhang H., Liu J. (2010). The role of arabidopsis *AtFes1A* in cytosolic Hsp70 stability and abiotic stress tolerance. Plant J..

[24] Yuan F., Leng B., Wang B. (2016). Progress in studying salt secretion from the salt glands in recretohalophytes: How do plants secrete salt?. Front. Plant Sci..

[25] Corpas F.J., Leterrier M., Valderrama R., Airaki M., Chaki M., Palma J.M., Barroso J.B. (2011). Nitric oxide imbalance provokes a nitrosative response in plants under abiotic stress. Plant Sci..

[26] Perumal J., Balasundaram G., Mahyuddin A.P., Choolani M., Olivo M. (2015). SERS-based quantitative detection of ovarian cancer prognostic factor haptoglobin. Int. J. Nanomed..

[27] Zhao B., Hao R., Wang Z., Zhang H., Hao Y., Zhang C., Liu Y. (2018). Green synthesis of multi-dimensional plasmonic coupling structures: Graphene oxide gapped gold nanostars for highly intensified surface enhanced raman scattering. Chem. Eng. J..

[28] Breitman M., Ruiz-Moreno S., Gil A.L. (2007). Experimental problems in Raman spectroscopy applied to pigment identification in mixtures. Spectrochim. Acta A Mol. Biomol. Spectrosc..

[29] Azbej T., Severs M.J., Rusk B.G., Bodnar R.J. (2007). In situ quantitative analysis of individual H_2_O–CO_2_ fluid inclusions by laser Raman spectroscopy. Chem. Geol..

[30] Mitchell B.L., Patwardhan A.J., Ngola S.M., Chan S., Sundararajan N. (2010). Experimental and statistical analysis methods for peptide detection using surface-enhanced Raman spectroscopy. J. Raman Spectrosc..

[31] And M.G., Feng M.L. (2003). SERS substrates fabricated by island lithography:  The silver/pyridine system. J. Phys. Chem. B.

[32] Kleinman S.L., Frontiera R.R., Henry A.I., Dieringer J.A., Van Duyne R.P. (2013). Creating, characterizing, and controlling chemistry with SERS hot spots. Phys. Chem. Chem. Phys..

[33] Yamamoto Y.S., Itoh T. (2016). Why and how do the shapes of surface-enhanced raman scattering spectra change? Recent progress from mechanistic studies. J. Raman Spectrosc..

[34] Schlucker S. (2014). Surface-enhanced raman spectroscopy: Concepts and chemical applications. Angew. Chem. Int. Ed. Engl..

[35] Panneerselvam R., Liu G.K., Wang Y.H., Liu J.Y., Ding S.Y., Li J.F., Wu D.Y., Tian Z.Q. (2017). Surface-enhanced raman spectroscopy: Bottlenecks and future directions. Chem. Commun..

[36] Itoh T., Yamamoto Y.S., Ozaki Y. (2017). Plasmon-enhanced spectroscopy of absorption and spontaneous emissions explained using cavity quantum optics. Chem. Soc. Rev..

[37] Pang S., Yang T., He L. (2016). Review of surface enhanced raman spectroscopic (sers) detection of synthetic chemical pesticides. TrAC Trends Anal. Chem..

[38] Cheng J.M., Liu Y.Z., Wang H.W. (2014). Effects of surface-modified nano-scale carbon black on Cu and Zn fractionations in contaminated soil. Int. J. Phytoremediat..

[39] Gasic K., Korban S.S., Madhava Rao K.V., Raghavendra A.S. (2006). Heavy metal stress. Physiology & Molecular Biology of Stress Tolerance in Plants.

[40] Yadav S.K. (2010). Heavy metals toxicity in plants: An overview on the role of glutathione and phytochelatins in heavy metal stress tolerance of plants. S. Afr. J. Bot..

[41] Sun B., Jiang X., Wang H., Song B., Zhu Y., Wang H., Su Y., He Y. (2015). Surface-enhancement Raman scattering sensing strategy for discriminating trace mercuric ion (II) from real water samples in sensitive, specific, recyclable, and reproducible manners. Anal. Chem..

[42] Lu Y.L., Zhong J., Yao G.H., Huang Q. (2018). A label-free sers approach to quantitative and selective detection of mercury (II) based on DNA aptamer-modified SiO_2_@Au core/shell nanoparticles. Sens. Actuator B-Chem..

[43] Makam P., Shilpa R., Kandjani A.E., Periasamy S.R., Sabri Y.M., Madhu C., Bhargava S.K., Govindaraju T. (2018). SERS and fluorescence-based ultrasensitive detection of mercury in water. Biosens. Bioelectron..

[44] Chen Y., Wu L.H., Chen Y.H., Bi N., Zheng X., Qi H.B., Qin M.H., Liao X., Zhang H.Q., Tian Y. (2012). Determination of mercury(II) by surface-enhanced Raman scattering spectroscopy based on thiol-functionalized silver nanoparticles. Microchim. Acta.

[45] Ma P.Y., Liang F.H., Yang Q.Q., Wang D., Sun Y., Wang X.H., Gao D.J., Song D.Q. (2014). Highly sensitive SERS probe for mercury(II) using cyclodextrin-protected silver nanoparticles functionalized with methimazole. Microchim. Acta.

[46] Xing N., Ji L.Z., Song J., Ma J.C., Li S.G., Ren Z.M., Xu F., Zhu J.P. (2017). Cadmium stress assessment based on the electrocardiogram characteristics of zebra fish (*Danio rerio*): QRS complex could play an important role. Aquat. Toxicol..

[47] Yin J., Wu T., Song J.B., Zhang Q., Liu S.Y., Xu R., Duan H.W. (2011). SERS-active nanoparticles for sensitive and selective detection of cadmium ion (Cd^2+^). Chem. Mater..

[48] Thatai S., Khurana P., Prasad S., Kumar D. (2015). Plasmonic detection of Cd^2+^ ions using surface-enhanced Raman scattering active core-shell nanocomposite. Talanta.

[49] Wang Y.L., Irudayaraj J. (2011). A SERS dnazyme biosensor for lead ion detection. Chem. Commun..

[50] Li C.N., Fan P.D., Liang A.H., Liu Q.Y., Jiang Z.L. (2018). Aptamer based determination of pb(II) by SERS and by exploiting the reduction of HAuCl_4_ by H_2_O_2_ as catalyzed by graphene oxide nanoribbons. Microchim. Acta.

[51] Frost M.S., Dempsey M.J., Whitehead D.E. (2015). Highly sensitive SERS detection of pb^2+^ ions in aqueous media using citrate functionalised gold nanoparticles. Sens. Actuator B-Chem..

[52] Sarfo D.K., Izake E.L., O’Mullane A.P., Ayoko G.A. (2018). Molecular recognition and detection of pb(II) ions in water by aminobenzo-18-crown-6 immobilised onto a nanostructured SERS substrate. Sens. Actuator B-Chem..

[53] Zhao L.Y., Gu W., Zhang C.L., Shi X.H., Xian Y.Z. (2016). In situ regulation nanoarchitecture of Au nanoparticles/reduced graphene oxide colloid for sensitive and selective SERS detection of lead ions. J. Colloid Interface Sci..

[54] Shi Y., Chen N., Su Y.Y., Wang H.Y., He Y. (2018). Silicon nanohybrid-based SERS chips armed with an internal standard for broad-range, sensitive and reproducible simultaneous quantification of lead(II) and mercury(II) in real systems. Nanoscale.

[55] Li J.L., Chen L.X., Lou T.T., Wang Y.Q. (2011). Highly sensitiveSERS detection of As^3+^ ions in aqueous media using glutathione functionalized silver nanoparticles. ACS Appl. Mater. Interfaces.

[56] Liu R., Sun J.F., Cao D., Zhang L.Q., Liu J.F., Jiang G.B. (2015). Fabrication of highly-specific SERS substrates by co-precipitation of functional nanomaterials during the self-sedimentation of silver nanowires into a nanoporous film. Chem. Commun..

[57] Song L.L., Mao K., Zhou X.D., Hu J.M. (2016). A novel biosensor based on Au@Ag core-shell nanoparticles for SERS detection of arsenic (III). Talanta.

[58] Xu Z.H., Hao J.M., Li F.S., Meng X.G. (2010). Surface-enhanced Raman spectroscopy of arsenate and arsenite using ag nanofilm prepared by modified mirror reaction. J. Colloid Interface Sci..

[59] Tao G.Q., Wang J. (2018). Gold nanorod@nanoparticle seed-SERS nanotags/graphene oxide plasmonic superstructured nanocomposities as an “on-off” SERS aptasensor. Carbon.

[60] Ndokoye P., Ke J., Liu J., Zhao Q.D., Li X.Y. (2014). l-cysteine-modified gold nanostars for SERS-based copper ions detection in aqueous media. Langmuir.

[61] Jin L.L., She G.W., Mu L.X., Shi W.S. (2016). Highly uniform indicator-mediated SERS sensor platform for the detection of Zn^2+^. RSC Adv..

[62] Zhao K.F., Song J., Fan H., Zhou S., Zhao M. (2010). Growth response to ionic and osmotic stress of NaCl in salt-tolerant and salt-sensitive maize. J. Integr. Plant Biol..

[63] Ying L.U., Shu S., Zhu W., Guo S. (2015). Effects of exogenous spermidine on the growth and quality of pakchoi seedlings under calcium nitrate stress. Acta Bot. Boreali-Occident. Sin..

[64] Han F.U., Shi Q.Q., Ming Z., Yu L., Biochemistry D.O., University J., Company D.B. (2016). Effect of copper sulphate stress during germination on the growth of mung bean and the intracellular density change response. J. Plant Physiol..

[65] Tsoutsi D., Montenegro J.M., Dommershausen F., Koert U., Liz-Marzán L.M., Parak W.J., Alvarez-Puebla R.A. (2011). Quantitative surface-enhanced Raman scattering ultradetection of atomic inorganic ions: The case of chloride. ACS Nano.

[66] Chi T.K.T., Tran H.T.T., Bui H.T.T., Dang T.Q., Nguyen L.Q. (2017). Determination of low level nitrate/nitrite contamination using SERS-active Ag/ITO substrates coupled to a self-designed Raman spectroscopy system. J. Sci. Adv. Mater..

[67] Ravindranath S.P., Kadam U.S., Thompson D.K., Irudayaraj J. (2012). Intracellularly grown gold nanoislands as SERS substrates for monitoring chromate, sulfate and nitrate localization sites in remediating bacteria biofilms by Raman chemical imaging. Anal. Chim. Acta.

[68] Shakir S.K., Irfan S., Akhtar B., Rehman S.U., Daud M.K., Taimur N., Azizullah A. (2018). Pesticide-induced oxidative stress and antioxidant responses in tomato (*Solanum lycopersicum*) seedlings. Ecotoxicology.

[69] Jiang Y., Sun D.-W., Pu H., Wei Q. (2018). Surface enhanced Raman spectroscopy (SERS): A novel reliable technique for rapid detection of common harmful chemical residues. Trends Food Sci. Technol..

[70] Chen J., Huang Y., Kannan P., Zhang L., Lin Z., Zhang J., Chen T., Guo L. (2016). Flexible and adhesive surface enhance Raman scattering active tape for rapid detection of pesticide residues in fruits and vegetables. Anal. Chem..

[71] Han X.X., Ji W., Zhao B., Ozaki Y. (2017). Semiconductor-enhanced Raman scattering: Active nanomaterials and applications. Nanoscale.

[72] Ji W., Zhao B., Ozaki Y. (2016). Semiconductor materials in analytical applications of surface-enhanced Raman scattering. J. Raman Spectrosc..

[73] Xu M.L., Gao Y., Han X.X., Zhao B. (2017). Detection of pesticide residues in food using surface-enhanced Raman spectroscopy: A review. J. Agric. Food Chem..

[74] Shi G., Wang M., Zhu Y., Wang Y., Ma W. (2018). Synthesis of flexible and stable SERS substrate based on Au nanofilms/cicada wing array for rapid detection of pesticide residues. Opt. Commun..

[75] Wijaya W., Pang S., Labuza T.P., He L. (2014). Rapid detection of acetamiprid in foods using surface-enhanced Raman spectroscopy (SERS). J. Sci. Food Agric..

[76] Weisman D., Alkio M., Colon-Carmona A. (2010). Transcriptional responses to polycyclic aromatic hydrocarbon-induced stress in *Arabidopsis thaliana* reveal the involvement of hormone and defense signaling pathways. BMC Plant Biol..

[77] Liu H., Weisman D., Tang L., Tan L., Zhang W.K., Wang Z.H., Huang Y.H., Lin W.X., Liu X.M., Colon-Carmona A. (2015). Stress signaling in response to polycyclic aromatic hydrocarbon exposure in *Arabidopsis thaliana* involves a nucleoside diphosphate kinase, NDPK-3. Planta.

[78] Limón-Pacheco J., Gonsebatt M.E. (2009). The role of antioxidants and antioxidant-related enzymes in protective responses to environmentally induced oxidative stress. Mutat. Res. Gen. Toxicol. Environ. Mutagen..

[79] Barreira L.A., Mudge S.M., Bebianno M.J. (2007). Oxidative stress in the Clam *Ruditapes decussatus* (linnaeus, 1758) in relation to polycyclic aromatic hydrocarbon body burden. Environ. Toxicol..

[80] Xie Y., Wang X., Han X., Song W., Ruan W., Liu J., Zhao B., Ozaki Y. (2011). Selective sers detection of each polycyclic aromatic hydrocarbon (PAH) in a mixture of five kinds of PAHs. J. Raman Spectrosc..

[81] Du J., Xu J., Sun Z., Jing C. (2016). Au nanoparticles grafted on Fe_3_O_4_ as effective sers substrates for label-free detection of the 16 EPA priority polycyclic aromatic hydrocarbons. Anal. Chim. Acta.

[82] Gu X., Tian S., Zhou Q., Adkins J., Gu Z., Li X., Zheng J. (2013). SERS detection of polycyclic aromatic hydrocarbons on a bowl-shaped silver cavity substrate. RSC Adv..

[83] Xie Y., Wang X., Han X., Xue X., Ji W., Qi Z., Liu J., Zhao B., Ozaki Y. (2010). Sensing of polycyclic aromatic hydrocarbons with cyclodextrin inclusion complexes on silver nanoparticles by surface-enhanced Raman scattering. Analyst.

[84] Kwon Y.-H., Sowoidnich K., Schmidt H., Kronfeldt H.-D. (2012). Application of calixarene to high active surface-enhanced Raman scattering (SERS) substrates suitable for in situ detection of polycyclic aromatic hydrocarbons (PAHs) in seawater. J. Raman Spectrosc..

[85] Sheng P., Wu S., Bao L., Wang X., Chen Z., Cai Q. (2012). Surface enhanced Raman scattering detecting polycyclic aromatic hydrocarbons with gold nanoparticle-modified TiO_2_ nanotube arrays. New J. Chem..

[86] Xu J., Du J., Jing C., Zhang Y., Cui J. (2014). Facile detection of polycyclic aromatic hydrocarbons by a surface-enhanced Raman scattering sensor based on the Au coffee ring effect. ACS Appl. Mater. Interfaces.

[87] Zhao H., Jin J., Tian W., Li R., Yu Z., Song W., Cong Q., Zhao B., Ozaki Y. (2015). Three-dimensional superhydrophobic surface-enhanced Raman spectroscopy substrate for sensitive detection of pollutants in real environments. J. Mater. Chem. A.

[88] Wang X., Hao W., Zhang H., Pan Y., Kang Y., Zhang X., Zou M., Tong P., Du Y. (2015). Analysis of polycyclic aromatic hydrocarbons in water with gold nanoparticles decorated hydrophobic porous polymer as surface-enhanced Raman spectroscopy substrate. Spectrochim. Acta A.

[89] Erofeeva E.A. (2018). Hormesis and paradoxical effects of pea (*Pisum sativum* L.) parameters upon exposure to formaldehyde in a wide range of doses. Ecotoxicology.

[90] Ye X., Ji Z., Wei C., McHale C.M., Ding S., Thomas R., Yang X., Zhang L. (2013). Inhaled formaldehyde induces DNA-protein crosslinks and oxidative stress in bone marrow and other distant organs of exposed mice. Environ. Mol. Mutagen..

[91] Feng L., Musto C.J., Suslick K.S. (2010). A simple and highly sensitive colorimetric detection method for gaseous formaldehyde. J. Am. Chem. Soc..

[92] De Oliveira F.S., Sousa E.T., de Andrade J.B. (2007). A sensitive flow analysis system for the fluorimetric determination of low levels of formaldehyde in alcoholic beverages. Talanta.

[93] Liu J.F., Peng J.F., Chi Y.G., Jiang G.B. (2005). Determination of formaldehyde in shiitake mushroom by ionic liquid-based liquid-phase microextraction coupled with liquid chromatography. Talanta.

[94] Qu W.G., Lu L.Q., Lin L., Xu A.W. (2012). A silver nanoparticle based surface enhanced resonance Raman scattering (SERS) probe for the ultrasensitive and selective detection of formaldehyde. Nanoscale.

[95] Zhang Z., Zhao C., Ma Y., Li G. (2014). Rapid analysis of trace volatile formaldehyde in aquatic products by derivatization reaction-based surface enhanced Raman spectroscopy. Analyst.

[96] Ma P., Liang F., Wang D., Yang Q., Ding Y., Yu Y., Gao D., Song D., Wang X. (2014). Ultrasensitive determination of formaldehyde in environmental waters and food samples after derivatization and using silver nanoparticle assisted SERS. Microchim. Acta.

[97] Lv Z.Y., Mei L.P., Chen W.Y., Feng J.J., Chen J.Y., Wang A.J. (2014). Shaped-controlled electrosynthesis of gold nanodendrites for highly selective and sensitive SERS detection of formaldehyde. Sens. Actuator B-Chem..

[98] Zhu Q.L., Sun L., Lian J.J., Gao X.L., Zhao L., Ding M.Y., Li J., Liang Y.C. (2016). The phospholipase c (*FgPLC1*) is involved in regulation of development, pathogenicity, and stress responses in *Fusarium graminearum*. Fungal Genet. Biol..

[99] Li H., Zhang F., Guo H., Zhu Y., Yuan J., Yang G., An L. (2013). Molecular characterization of hepcidin gene in common carp (*Cyprinus carpio* L.) and its expression pattern responding to bacterial challenge. Fish Shellfish Immunol..

[100] Yang G., Guo H., Li H., Shan S., Zhang X., Rombout J.H., An L. (2014). Molecular characterization of LEAP-2 cDNA in common carp (*Cyprinus carpio* L.) and the differential expression upon a *Vibrio anguillarum* stimulus; indications for a significant immune role in skin. Fish Shellfish Immunol..

[101] Shan S.J., Liu D.Z., Wang L., Zhu Y.Y., Zhang F.M., Li T., An L.G., Yang G.W. (2015). Identification and expression analysis of *irak1* gene in common carp *Cyprinus carpio* L.: Indications for a role of antibacterial and antiviral immunity. J. Fish Biol..

[102] Zhang F., Huang Y.H., Liu S.Z., Zhang L., Li B.T., Zhao X.X., Fu Y., Liu J.J., Zhang X.X. (2013). *Pseudomonas reactans*, a Bacterial strain isolated from the intestinal flora of *Blattella germanica* with anti-*Beauveria bassiana* activity. Environ. Entomol..

[103] Wang F., Yang H.J., He H.B., Wang C.F., Gao Y.D., Zhong Q.F., Wang X.H., Zeng Y.J. (2011). Study on the hemolysin phenotype and the genetype distribution of staphyloccocus aureus caused bovine mastitis in shandong dairy farms. Int. J. Appl. Res. Vet. Med..

[104] Wang J., Wu X., Wang C., Shao N., Dong P., Xiao R., Wang S. (2015). Magnetically assisted surface-enhanced Raman spectroscopy for the detection of *Staphylococcus aureus* based on aptamer recognition. ACS Appl. Mater. Interfaces.

[105] Zhang H., Ma X., Liu Y., Duan N., Wu S., Wang Z., Xu B. (2015). Gold nanoparticles enhanced SERS aptasensor for the simultaneous detection of *Salmonella typhimurium* and *Staphylococcus aureus*. Biosens. Bioelectron..

[106] Zhang A.W., Tao G.Q., Wang J. (2018). Assembly of bioconjugated rod-nanotags and multilayer plasmonic nanorod-array for ultrasensitive SERS detection of *S. aureus* bacteria. J. Nanopart. Res..

[107] Lin C.C., Yang Y.M., Liao P.H., Chen D.W., Lin H.P., Chang H.C. (2014). A filter-like AuNPs@MS SERS substrate for *Staphylococcus aureus* detection. Biosens. Bioelectron..

[108] Kowalska A.A., Kaminska A., Adamkiewicz W., Witkowska E., Tkacz M. (2015). Novel highly sensitive Cu-based SERS platforms for biosensing applications. J. Raman Spectrosc..

[109] Duan N., Shen M.F., Wu S.J., Zhao C.X., Ma X.Y., Wang Z.P. (2017). Graphene oxide wrapped Fe_3_O_4_@Au nanostructures as substrates for aptamer-based detection of *Vibrio parahaemolyticus* by surface-enhanced Raman spectroscopy. Microchim. Acta.

[110] Xu J.J., Turner J.W., Idso M., Biryukov S.V., Rognstad L., Gong H., Trainer V.L., Wells M.L., Strom M.S., Yu Q.M. (2013). In situ strain-level detection and identification of *Vibrio parahaemolyticus* using surface-enhanced Raman spectroscopy. Anal. Chem..

[111] He L.L., Deen B.D., Pagel A.H., Diez-Gonzalez F., Labuza T.P. (2013). Concentration, detection and discrimination of *Bacillus anthracis* spores in orange juice using aptamer based surface enhanced Raman spectroscopy. Analyst.

[112] Zhang B.H., Wang H.S., Lu L.H., Ai K.L., Zhang G., Cheng X.L. (2008). Large-area silver-coated silicon nanowire arrays for molecular sensing using surface-enhanced Raman spectroscopy. Adv. Funct. Mater..

[113] Farquharson S., Shende C., Smith W., Huang H., Inscore F., Sengupta A., Sperry J., Sickler T., Prugh A., Guicheteau J. (2014). Selective detection of 1000 B. *anthracis* spores within 15 minutes using a peptide functionalized SERS assay. Analyst.

[114] Cheung M., Lee W.W.Y., Cowcher D.P., Goodacre R., Bell S.E.J. (2016). SERS of meso-droplets supported on superhydrophobic wires allows exquisitely sensitive detection of dipicolinic acid, an anthrax biomarker, considerably below the infective close. Chem. Commun..

[115] Bai X.R., Zeng Y., Zhou X.D., Wang X.H., Shen A.G., Hu J.M. (2017). Environmentally safe mercury(II) ions aided zero-background and ultrasensitive SERS detection of dipicolinic acid. Anal. Chem..

[116] Hou P., Zhao G., He C., Wang H., He H. (2018). Biopanning of polypeptides binding to bovine ephemeral fever virus G_1_ protein from phage display peptide library. BMC Vet. Res..

[117] Hou P., Wang H., Zhao G., He C., He H. (2017). Rapid detection of infectious bovine Rhinotracheitis virus using recombinase polymerase amplification assays. BMC Vet. Res..

[118] Liang J.W., Tian F.L., Lan Z.R., Huang B., Zhuang W.Z. (2010). Selection characterization on overlapping reading frame of multiple-protein-encoding P gene in Newcastle disease virus. Vet. Microbiol..

[119] Yu J., Wu J.Q., Zhang Y.Y., Guo L.H., Cong X.Y., Du Y.J., Li J., Sun W.B., Shi J.L., Peng J. (2012). Concurrent highly pathogenic porcine reproductive and respiratory syndrome virus infection accelerates *Haemophilus parasuis* infection in conventional pigs. Vet. Microbiol..

[120] Shan S., Qi C., Zhu Y., Li H., An L., Yang G. (2016). Expression profile of carp IFN correlate with the up-regulation of interferon regulatory factor-1 (IRF-1) in vivo and in vitro: The pivotal molecules in antiviral defense. Fish Shellfish Immunol..

[121] Zhu Y., Qi C., Shan S., Zhang F., Li H., An L., Yang G. (2016). Characterization of common carp (*Cyprinus carpio* L.) interferon regulatory factor 5 (IRF5) and its expression in response to viral and bacterial challenges. BMC Vet. Res..

[122] Li T., Li H., Peng S., Zhang F., An L., Yang G. (2017). Molecular characterization and expression pattern of X box-binding protein-1 (XBP1) in common carp (*Cyprinus carpio* L.): Indications for a role of XBP1 in antibacterial and antiviral immunity. Fish Shellfish Immunol..

[123] Zheng S., Wu X., Zhang L., Xin C., Liu Y., Shi J., Peng Z., Xu S., Fu F., Yu J. (2017). The occurrence of porcine circovirus 3 without clinical infection signs in shandong province. Transbound. Emerg. Dis..

[124] Sivashanmugan K., Liao J.D., You J.W., Wu C.L. (2013). Focused-ion-beam-fabricated Au/Ag multilayered nanorod array as SERS-active substrate for virus strain detection. Sens. Actuator B-Chem..

[125] Lim J.Y., Nam J.S., Yang S.E., Shin H., Jang Y.H., Bae G.U., Kang T., Lim K.I., Choi Y. (2015). Identification of newly emerging influenza viruses by surface-enhanced Raman spectroscopy. Anal. Chem..

[126] Sun Y., Xu L., Zhang F.D., Song Z.G., Hu Y.W., Ji Y.J., Shen J.Y., Li B., Lu H.Z., Yang H.F. (2017). A promising magnetic SERS immunosensor for sensitive detection of avian influenza virus. Biosens. Bioelectron..

[127] Luo Z.H., Li W.T., Lu D.L., Chen K., He Q.G., Han H.Y., Zou M.Q. (2013). A SERS-based immunoassay for porcine circovirus type 2 using multi-branched gold nanoparticles. Microchim. Acta.

[128] Serra A., Manno D., Filippo E., Buccolieri A., Urso E., Rizzello A., Maffia M. (2011). SERS based optical sensor to detect prion protein in neurodegenerate living cells. Sens. Actuator B-Chem..

[129] Ashmore M., Toet S., Emberson L. (2010). Ozone-a significant threat to future world food production?. New Phytol..

[130] Pina J.M., Moraes R.M. (2010). Gas exchange, antioxidants and foliar injuries in saplings of a tropical woody species exposed to ozone. Ecotoxicol. Environ. Saf..

[131] Tausz M., Grulke N.E., Wieser G. (2007). Defense and avoidance of ozone under global change. Environ. Pollut..

[132] Zhang X., Lin C., Liu Q., Liang A. (2014). An ultrasensitive SERS method for the determination of ozone using a nanogold sol as substrate and rhodamines as probe. RSC Adv..

[133] Wu B., Shao H., Wang Z., Hu Y., Tang Y.J., Jun Y.S. (2010). Viability and metal reduction of shewanella oneidensis MR-1 under CO_2_ stress: Implications for ecological effects of CO_2_ leakage from geologic CO_2_ sequestration. Environ. Sci. Technol..

[134] Iwasaki I., Kurano N., Miyachi S. (1996). Effects of high-CO_2_ stress on photosystem II in a green alga, chlorococcum littorale, which has a tolerance to high CO_2_. J. Photochem. Photobiol. B.

[135] Lust M., Pucci A., Akemann W., Otto A. (2008). SERS of CO_2_ on cold-deposited Cu: An electronic effect at a minority of surface sites. J. Phys. Chem. C.

[136] Bontempi N., Carletti L., De Angelis C., Alessandri I. (2016). Plasmon-free SERS detection of environmental CO_2_ on TiO_2_ surfaces. Nanoscale.

[137] Jun-Cheol M., Woncheol Y., Sungdon L., Song K.T., Byung-Moo L. (2014). Differentially expressed genes and in silico analysis in response to ozone (O_3_) stress of soybean leaves. Aust. J. Sci..

[138] Li K., Pang C.H., Ding F., Sui N., Feng Z.T., Wang B.S. (2012). Overexpression of *Suaeda salsa* stroma ascorbate peroxidase in *Arabidopsis* chloroplasts enhances salt tolerance of plants. S. Afr. J. Bot..

[139] Chen Q.F., Rao Y.Y., Ma X.Y., Dong J.A., Qian W.P. (2011). Raman spectroscopy for hydrogen peroxide scavenging activity assay using gold nanoshell precursor nanocomposites as SERS probes. Anal. Methods.

[140] Chen Y.C., Hsu J.H., Lin Y.G., Hsu Y.K. (2017). Silver nanowires on coffee filter as dual-sensing functionality for efficient and low-cost SERS substrate and electrochemical detection. Sens. Actuator B-Chem..

[141] Chen Y.C., Hsu J.H., Hsu Y.K. (2018). Branched silver nanowires on fluorine-doped tin oxide glass for simultaneous amperometric detection of H_2_O_2_ and of 4-aminothiophenol by SERS. Microchim. Acta.

[142] Qin L., Li X., Kang S.-Z., Mu J. (2015). Gold nanoparticles conjugated dopamine as sensing platform for SERS detection. Colloids Surf. B. Biointerfaces.

[143] Dong J., Guo G.M., Xie W., Li Y., Zhang M.Y., Qian W.P. (2015). Free radical-quenched SERS probes for detecting H_2_O_2_ and glucose. Analyst.

[144] Pang C.H., Li K., Wang B. (2011). Overexpression of *SsCHLAPXs* confers protection against oxidative stress induced by high light in transgenic *Arabidopsis thaliana*. Physiol. Plant..

[145] Feng Z.T., Deng Y.Q., Fan H., Sun Q.J., Sui N., Wang B.S. (2014). Effects of NaCl stress on the growth and photosynthetic characteristics of *Ulmus pumila* L. seedlings in sand culture. Photosynthetica.

[146] Han N., Lan W., He X., Shao Q., Wang B., Zhao X. (2011). Expression of a *Suaeda salsa* vacuolar H^+^/Ca^2+^ transporter gene in *Arabidopsis* contributes to physiological changes in salinity. Plant Mol. Biol. Rep..

[147] Sun W., Li Y., Zhao Y., Zhang H. (2014). The TsnsLTP4, a nonspecific lipid transfer protein involved in wax deposition and stress tolerance. Plant Mol. Biol. Rep..

[148] Sun G.J., Pan J., Liu K.C., Wang S.F., Wang X., Wang X.M. (2012). Molecular cloning and expression analysis of P-selectin glycoprotein ligand-1 from zebrafish (*Danio rerio*). Fish Physiol. Biochem..

[149] Sui N., Li M., Li K., Song J., Wang B.S. (2010). Increase in unsaturated fatty acids in membrane lipids of *Suaeda salsa* L. Enhances protection of photosystem ii under high salinity. Photosynthetica.

[150] Das S.K., Patra J.K., Thatoi H. (2016). Antioxidative response to abiotic and biotic stresses in mangrove plants: A review. Int. Rev. Hydrobiol..

[151] Chen M., Zhang W.H., Lv Z.W., Zhang S.L., Hidema J., Shi F.M., Liu L.L. (2013). Abscisic acid is involved in the response of *Arabidopsis* mutant *sad2-1* to ultraviolet-B radiation by enhancing antioxidant enzymes. S. Afr. J. Bot..

[152] Cao S., Du X.H., Li L.H., Liu Y.D., Zhang L., Pan X., Li Y., Li H., Lu H. (2017). Overexpression of *populus tomentosa* cytosolic ascorbate peroxidase enhances abiotic stress tolerance in tobacco plants. Russ. J. Plant Physiol..

[153] Qi Y.-C., Wang F.-F., Zhang H., Liu W.-Q. (2009). Overexpression of *Suadea salsa* S-adenosylmethionine synthetase gene promotes salt tolerance in transgenic tobacco. Acta Physiol. Plant.

[154] Qi Y.C., Liu W.Q., Qiu L.Y., Zhang S.M., Ma L., Zhang H. (2010). Overexpression of glutathione *S*-transferase gene increases salt tolerance of arabidopsis. Russ. J. Plant Physiol..

[155] Zhang S., Song J., Wang H., Feng G. (2010). Effect of salinity on seed germination, ion content and photosynthesis of cotyledons in halophytes or xerophyte growing in central Asia. J. Plant Ecol..

[156] Jiang C., Li Y., Liu C., Qiu L., Li Z. (2016). A general and versatile fluorescence turn-on assay for detecting the activity of protein tyrosine kinases based on phosphorylation-inhibited tyrosyl oxidation. Chem. Commun..

[157] Cai H., Huang B., Lin R., Xu P., Liu Y., Zhao Y. (2018). A “turn-off” SERS assay for kinase detection based on arginine N-phosphorylation process. Talanta.

[158] Bjerneld E.J., Földes-Papp Z., Käll M.A., Rigler R. (2010). Single-molecule surface-enhanced Raman and fluorescence correlation spectroscopy of horseradish peroxidase. J. Phys. Chem. B.

[159] Kahraman M., Sur I., Culha M. (2010). Label-free detection of proteins from self-assembled protein-silver nanoparticle structures using surface-enhanced Raman scattering. Anal. Chem..

[160] Giorgis F., Descrovi E., Chiodoni A., Froner E., Scarpa M., Venturello A., Geobaldo F. (2008). Porous silicon as efficient surface enhanced Raman scattering (SERS) substrate. Appl. Surf. Sci..

[161] Cottat M., D’Andrea C., Yasukuni R., Malashikhina N., Grinyte R., Lidgi-Guigui N., Fazio B., Sutton A., Oudar O., Charnaux N. (2015). High sensitivity, high selectivity SERS detection of MnSOD using optical nanoantennas functionalized with aptamers. J. Phys. Chem. C.

[162] Li X., Liu Y., Chen M., Song Y.P., Song J., Wang B.S., Feng G. (2012). Relationships between ion and chlorophyll accumulation in seeds and adaptation to saline environments in *Suaeda salsa* populations. Plant Biosyst..

[163] Lian W.N., Shiue J., Wang H.H., Hong W.C., Shih P.H., Hsu C.K., Huang C.Y., Hsing C.R., Wei C.M., Wang J.K. (2015). Rapid detection of copper chlorophyll in vegetable oils based on surface-enhanced Raman spectroscopy. Food Addit. Contam. A.

[164] Aguilar-Hernández I., Afseth N.K., López-Luke T., Contreras-Torres F.F., Wold J.P., Ornelas-Soto N. (2017). Surface enhanced Raman spectroscopy of phenolic antioxidants: A systematic evaluation of ferulic acid, p-coumaric acid, caffeic acid and sinapic acid. Vib. Spectrosc..

[165] Jurasekova Z., Domingo C., Garcia-Ramos J.V., Sanchez-Cortes S. (2014). Effect of pH on the chemical modification of quercetin and structurally related flavonoids characterized by optical (UV-visible and Raman) spectroscopy. Phys. Chem. Chem. Phys..

[166] Huang C.C., Chen W. (2018). A SERS method with attomolar sensitivity: A case study with the flavonoid catechin. Microchim. Acta.

[167] Liu F., Yang Y., Gao J., Ma C., Bi Y. (2018). A comparative transcriptome analysis of a wild purple potato and its red mutant provides insight into the mechanism of anthocyanin transformation. PLoS ONE.

[168] Song J., Wang B. (2015). Using euhalophytes to understand salt tolerance and to develop saline agriculture: *Suaeda salsa* as a promising model. Ann. Bot..

[169] Kong X., Wang T., Li W., Tang W., Zhang D., Dong H. (2016). Exogenous nitric oxide delays salt-induced leaf senescence in cotton (*Gossypium hirsutum* L.). Acta Physiol. Plant..

[170] Yang Z., Wang Y., Wei X., Zhao X., Wang B., Sui N. (2017). Transcription profiles of genes related to hormonal regulations under salt stress in sweet sorghum. Plant Mol. Biol. Rep..

[171] Zhang L., Zhang X., Fan S. (2017). Meta-analysis of salt-related gene expression profiles identifies common signatures of salt stress responses in arabidopsis. Plant Syst. Evol..

[172] Zhang Y., Kong X., Dai J., Luo Z., Li Z., Lu H., Xu S., Tang W., Zhang D., Li W. (2017). Global gene expression in cotton (*Gossypium hirsutum* L.) leaves to waterlogging stress. PLoS ONE.

[173] Wang J., Zhang Q., Cui F., Hou L., Zhao S., Xia H., Qiu J., Li T., Zhang Y., Wang X. (2017). Genome-wide analysis of gene expression provides new insights into cold responses in *Thellungiella salsuginea*. Front. Plant Sci..

[174] Yuan F., Lyu M.J., Leng B.Y., Zhu X.G., Wang B.S. (2016). The transcriptome of NaCl-treated *Limonium bicolor* leaves reveals the genes controlling salt secretion of salt gland. Plant Mol. Biol..

[175] Song J., Zhou J., Zhao W., Xu H., Wang F., Xu Y., Wang L., Tian C. (2016). Effects of salinity and nitrate on production and germination of dimorphic seeds applied both through the mother plant and exogenously during germination in *Suaeda salsa*. Plant Spec. Biol..

[176] Wang F., Xu Y.G., Wang S., Shi W., Liu R., Feng G., Song J. (2015). Salinity affects production and salt tolerance of dimorphic seeds of *Suaeda salsa*. Plant Physiol. Biochem..

[177] Zhao B., Dai A., Wei H., Yang S., Wang B., Jiang N., Feng X. (2016). *Arabidopsis* KLU homologue GmCYP78A72 regulates seed size in soybean. Plant Mol. Biol..

[178] Liu W., Ji S., Fang X., Wang Q., Li Z., Yao F., Hou L., Dai S. (2013). Protein kinase LTRPK1 influences cold adaptation and microtubule stability in rice. J. Plant Growth Regul..

[179] Novak O., Napier R., Ljung K. (2017). Zooming in on plant hormone analysis: Tissue- and cell-specific approaches. Annu. Rev. Plant Biol..

[180] Zhou J., Liu Q., Zhang F., Wang Y., Zhang S., Cheng H., Yan L., Li L., Chen F., Xie X. (2014). Overexpression of OsPIL15, a phytochrome-interacting factor-like protein gene, represses etiolated seedling growth in rice. J. Integr. Plant Biol..

[181] Xu Y.G., Liu R., Sui N., Shi W., Wang L., Tian C., Song J. (2016). Changes in endogenous hormones and seed-coat phenolics during seed storage of two *Suaeda salsa* populations. Aust. J. Bot..

[182] Meng X., Yang D., Li X., Zhao S., Sui N., Meng Q. (2015). Physiological changes in fruit ripening caused by overexpression of tomato *SLAN*2, an R2R3-MYB factor. Plant Physiol. Biochem..

[183] Wu X.X., He J., Chen J.L., Yang S.J., Zha D.S. (2014). Alleviation of exogenous 6-benzyladenine on two genotypes of eggplant (*Solanum melongena* Mill.) growth under salt stress. Protoplasma.

[184] Zhang P., Wang L.M., Zheng D.W., Lin T.F., Wei X.D., Liu X.Y., Wang H.Q. (2018). Surface-enhanced Raman spectroscopic analysis of N^6^-benzylaminopurine residue quantity in sprouts with gold nanoparticles. J. Environ. Sci. Health B.

[185] Wang F., Gu X., Zheng C., Dong F., Zhang L., Cai Y., You Z., You J., Du S., Zhang Z. (2017). Ehrlich reaction evoked multiple spectral resonances and gold nanoparticle hotspots for raman detection of plant hormone. Anal. Chem..

[186] Miao C., Zhang Z., Liu M., Chen Q., Hua Y., Chen X. (2017). In situ fabrication of label-free optical sensing paper strips for the rapid surface-enhanced Raman scattering (SERS) detection of brassinosteroids in plant tissues. Talanta.

[187] Chen T., Yuan F., Song J., Wang B. (2016). Nitric oxide participates in waterlogging tolerance through enhanced adventitious root formation in the euhalophyte *Suaeda salsa*. Funct. Plant Biol..

[188] Cui J., Hu K., Sun J.J., Qu L.L., Li D.W. (2016). SERS nanoprobes for the monitoring of endogenous nitric oxide in living cells. Biosens. Bioelectron..

[189] Xu Q., Liu W., Li L., Zhou F., Zhou J., Tian Y. (2017). Ratiometric SERS imaging and selective biosensing of nitric oxide in live cells based on trisoctahedral gold nanostructures. Chem. Commun..

[190] Zhang Z., Han X., Wang Z., Yang Z., Zhang W., Li J., Yang H., Ling X.Y., Xing B. (2018). A live bacteria SERS platform for the in situ monitoring of nitric oxide release from a single mrsa. Chem. Commun..

[191] Bobba K.N., Saranya G., Alex S.M., Velusamy N., Maiti K.K., Bhuniya S. (2018). SERS-active multi-channel fluorescent probe for NO: Guide to discriminate intracellular biothiols. Sens. Actuator B-Chem..

[192] Sui N., Han G. (2014). Salt-induced photoinhibition of PSII is alleviated in halophyte *Thellungiella halophila* by increases of unsaturated fatty acids in membrane lipids. Acta Physiol. Plant..

[193] Tang G.Y., Wei L.Q., Liu Z.J., Bi Y.P., Shan L. (2012). Ectopic expression of peanut acyl carrier protein in tobacco alters fatty acid composition in the leaf and resistance to cold stress. Biol. Plant..

[194] Guo Y., Jia W., Song J., Wang D., Chen M., Wang B. (2012). *Thellungilla halophila* is more adaptive to salinity than *Arabidopsis thaliana* at stages of seed germination and seedling establishment. Acta Physiol. Plant..

[195] Ding F., Chen M., Sui N., Wang B.S. (2010). Ca^2+^ significantly enhanced development and salt-secretion rate of salt glands of *Limonium bicolor* under NaCl treatment. S. Afr. J. Bot..

[196] Sun Y.L., Li F., Su N., Sun X.L., Zhao S.J., Meng Q.W. (2010). The increase in unsaturation of fatty acids of phosphatidylglycerol in thylakoid membrane enhanced salt tolerance in tomato. Photosynthetica.

[197] Dong W., Zhang Y., Zhang B., Wang X. (2013). Rapid prediction of fatty acid composition of vegetable oil by Raman spectroscopy coupled with least squares support vector machines. J. Raman Spectrosc..

[198] He X.N., Gao Y., Mahjouri-Samani M., Black P.N., Allen J., Mitchell M., Xiong W., Zhou Y.S., Jiang L., Lu Y.F. (2012). Surface-enhanced Raman spectroscopy using gold-coated horizontally aligned carbon nanotubes. Nanotechnology.

[199] Šimáková P., Kočišová E., Procházka M. (2013). Sensitive Raman spectroscopy of lipids based on drop deposition using DCDR and SERS. J. Raman Spectrosc..

[200] Sweetenham C.S., Larraona-Puy M., Notingher I. (2011). Simultaneous surface-enhanced Raman spectroscopy (SERS) and atomic force microscopy (AFM) for label-free physicochemical analysis of lipid bilayers. Appl. Spectrosc..

[201] Kong L., Xie Y., Hu L., Si J., Wang Z. (2017). Excessive nitrogen application dampens antioxidant capacity and grain filling in wheat as revealed by metabolic and physiological analyses. Sci. Rep..

[202] He Y., Li Y., Cui L., Xie L., Zheng C., Zhou G., Zhou J., Xie X. (2016). Phytochrome B negatively affects cold tolerance by regulating *OsDREB1* gene expression through phytochrome interacting factor-like protein OsPIL16 in rice. Front Plant Sci..

[203] Zhou J.C., Fu T.T., Sui N., Guo J.R., Feng G., Fan J.L., Song J. (2014). The role of salinity in seed maturation of the euhalophyte *Suaeda salsa*. Plant Biosyst..

[204] Zhang D., Haputhanthri R., Ansar S.M., Vangala K., De Silva H.I., Sygula A., Saebo S., Pittman C.U. (2010). Ultrasensitive detection of malondialdehyde with surface-enhanced Raman spectroscopy. Anal. Bioanal. Chem..

[205] Liu J., Zhang F., Zhou J.J., Chen F., Wang B.S., Xie X.Z. (2012). Phytochrome B control of total leaf area and stomatal density affects drought tolerance in rice. Plant Mol. Biol..

[206] Han G.L., Wang M.J., Yuan F., Sui N., Song J., Wang B.S. (2014). The CCCH zinc finger protein gene *ATZFP1* improves salt resistance in *Arabidopsis thaliana*. Plant Mol. Biol..

[207] Guo J.R., Suo S.S., Wang B.S. (2015). Sodium chloride improves seed vigour of the euhalophyte *Suaeda salsa*. Seed Sci. Res..

[208] Sun Z.B., Qi X.Y., Wang Z.L., Li P.H., Wu C.X., Zhang H., Zhao Y.X. (2013). Overexpression of *TsGOLS2*, a galactinol synthase, in *Arabidopsis thaliana* enhances tolerance to high salinity and osmotic stresses. Plant Physiol. Biochem..

[209] Mary Y.S., Ushakumari L., Harikumar B., Varghese H.T., Panicker C.Y. (2009). FT-IR, FT-raman and SERS spectra of L-proline. J. Iran. Chem. Soc..

[210] Carcamo J.J., Aliaga A.E., Clavijo E., Garrido C., Gomez-Jeria J.S., Campos-Vallette M.M. (2012). Proline and hydroxyproline deposited on silver nanoparticles. A Raman, SERS and theoretical study. J. Raman Spectrosc..

[211] Kong K.V., Lam Z., Lau W.K., Leong W.K., Olivo M. (2013). A transition metal carbonyl probe for use in a highly specific and sensitive SERS-based assay for glucose. J. Am. Chem. Soc..

[212] Sun D., Qi G.H., Xu S.P., Xu W.Q. (2016). Construction of highly sensitive surface-enhanced raman scattering (SERS) nanosensor aimed for the testing of glucose in urine. RSC Adv..

[213] Sun F., Bai T., Zhang L., Ellamenye J.R., Liu S., Nowinski A.K., Jiang S., Yu Q. (2014). Sensitive and fast detection of fructose in complex media via symmetry breaking and signal amplification using surface-enhanced Raman spectroscopy. Anal. Chem..

[214] Qi G.H., Jia K.Q., Fu C.C., Xu S.P., Xu W.Q. (2015). A highly sensitive SERS sensor for quantitative analysis of glucose based on the chemical etching of silver nanoparticles. J. Opt..

[215] Gu X., Wang H., Schultz Z.D., Camden J.P. (2016). Sensing glucose in urine and serum and hydrogen peroxide in living cells by use of a novel boronate nanoprobe based on surface-enhanced Raman spectroscopy. Anal. Chem..

[216] Fu C., Jin S., Oh J., Xu S.P., Jung Y.M. (2017). Facile detection of glucose in human serum employing silver-ion-guided surface-enhanced Raman spectroscopy signal amplification. Analyst.

[217] Cai J., Huang J., Ge M., Iocozzia J., Lin Z., Zhang K.Q., Lai Y. (2017). Immobilization of Pt nanoparticles via rapid and reusable electropolymerization of dopamine on TiO_2_ nanotube arrays for reversible SERS substrates and nonenzymatic glucose sensors. Small.

[218] Bhardwaj V., Srinivasan S., J McGoron A. (2013). AgNPs-based label-free colloidal SERS nanosensor for the rapid and sensitive detection of stress-proteins expressed in response to environmental-toxins. J. Biosens. Bioelectron..

[219] Ma S., Huang Q. (2015). A SERS study of oxidation of glutathione under plasma irradiation. RSC Adv..

